# Molecular evidence from xenacoelomorph gonopore formation supports homology with the bilaterian anus

**DOI:** 10.1038/s41559-025-02866-6

**Published:** 2025-10-24

**Authors:** Carmen Andrikou, Kevin Pang, Aina Børve, Tsai-Ming Lu, Andreas Hejnol

**Affiliations:** 1 Friedrich Schiller University Jena, Institute of Zoology and Evolutionary Research, Jena, Germany; 2 University of Bergen, Department of Biological Sciences, Bergen, Norway; 3 Sars International Centre for Marine Molecular Biology, University of Bergen, Bergen, Norway; 4 Institute of Cellular and Organismic Biology, Academia Sinica, Taipei, Taiwan

**Keywords:** Evolutionary developmental biology, Animal physiology

## Abstract

The bilaterian through gut with an anal opening is a major evolutionary innovation in animals. It facilitates effective food processing, which allows animals to grow to a larger body size. However, because non-bilaterian animals (such as cnidarians) lack a through gut, the evolution of the anus in bilaterians (such as insects and humans) remains unresolved. The formation of the bilaterian hindgut is driven by the spatial expression of several transcription factors (for example, Caudal and Brachyury) under the control of Wnt signalling. Here we show that this bilaterian ‘hindgut‘ molecular signature is expressed around the male gonopore of several xenacoelomorphs, which possess a blind gut without an anal opening. Since xenacoelomorphs are the potential sister group to all remaining Bilateria, our results suggest that the bilaterian anus shares a deep evolutionary relationship with the xenacoelomorph male gonopore. We therefore propose that the bilaterian anus evolved from a male gonopore that secondarily connected to the digestive tract.

## Abstract (web summary)

The evolutionary origin of the bilaterian gut with two openings is debated. Expression in xenacoelomorphs, the sister group to bilaterians, of genes involved in bilaterian gut patterning suggests that the xenacoelomorph gonopore is homologous to the bilaterian anus.

## Main

**Fig. 1:**
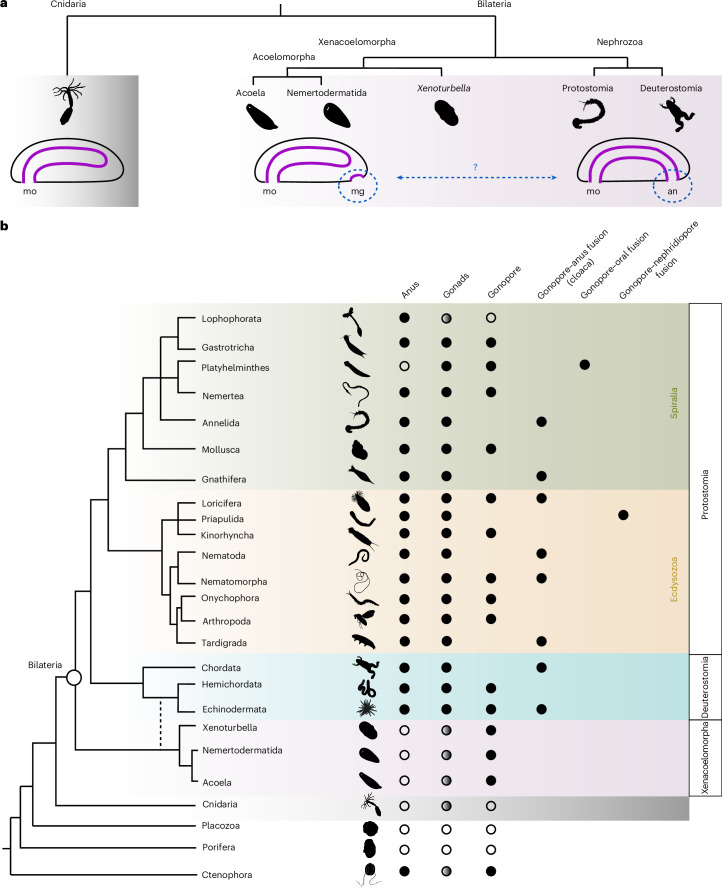
The hypothetical evolutionary transition from a blind gut to a through gut. **a**, Cnidarians possess a blind gut with a mouth, while nephrozoans (protostomes and deuterostomes) have a through gut with a mouth and an anus. Xenacoelomorphs, the probable sister group to the remaining Bilateria, are characterized by a blind gut with a mouth and a male gonopore. Whether the emergence of nephrozoan anus is evolutionarily related to the xenacoelomorph male gonopore remains unsolved. **b**, Phylogenetic distribution of the anus, gonopore, gonads and cloaca. Blind digestive systems (gut without anal opening) are found in non-bilaterians, Xenacoelomorphs and Platyhelminthes. Gonopores are found in members of all animal groups, while cloaca is only witnessed in members of protostomes and deuterostomes. Gonopores can vary in their location and morphology; they can be placed posterior-ventrally (for example, in arthropods^93,94^), ventro-laterally (for example, in nemerteans^95^), anterior-ventrally (for example, in *Hofsteniida* (acoelomorphs)^70^) or even dorsally (for example, in Catenulida (Platyhelminthes)^96,97^). Gonopores can also exist either as separate, sometimes even segmental, entities (for example, in nemerteans, arthropods and acoelomorphs)^53,93–95^ or in connection to either the posterior portion of the digestive system (anus) forming a common opening called cloaca (for example, in chordates, loriciferans, rotifers, holothurians, nematomorphs and tardigrades)^98–104^ or the anterior portion of the digestive system (mouth) (for example, in Lecithoepitheliata and Prolecithophora (Platyhelminthes)^96,97^). Non-bilaterians lack gonopores (with the exception of Ctenophora^49^) and they release their gametes either via the oscula (for example, in Porifera^48^) or through the mouth opening/transient openings of the body (for example, in Cnidaria^47^). White circles show the absence of gonads and grey circles indicate that epithelial lined gonads are not present in these species. an, anus; mg, male gonopore; mo, mouth. Credit: Silhouettes from phylopic.org under a Creative Commons license: *Diopatra cuprea* and *Gordiacea* (CC BY 4.0), *Xenopus laevis*, *Priapulus caudatus* and *Pomatias elegans* (CC BY 3.0), *Pleurobrachia*, *Trichoplax adhaerens* and *Notospermus* (CC BY-SA 3.0); the remaining icons are in the public domain.

The evolution of a through gut—a digestive tube with two openings, the mouth and the anus—allowed the unidirectional movement of the food through a regionalized gut and therefore the efficient processing of nutrients. However, a through gut is only found in bilaterians, while the closely related Cnidaria possess a blind gut with only one opening (Fig. 1). How and when the anus emerged during evolution is therefore crucial for understanding the evolution of animal macrofauna and it has been the subject of debates for more than 100 years (Fig. 1a)^1–6^. During bilaterian gastrulation the primitive digestive tube compartmentalizes along its anterior–posterior (AP) axis in the distinct sections of foregut, midgut and hindgut. These territories will eventually give rise to the specialized tissues of the adult gut: the foregut will form the oesophagus and stomach, the midgut will form the small intestine and the hindgut will form the large intestine and part of the anal canal. The regionalization process of the gut primordia is vital for its proper function and is controlled by the localized expression of several transcription factors, some of which are shown to have not only a conserved expression domain but also a conserved pivotal role^7,8^.

In particular, studies on the molecular patterning of the bilaterian hindgut development, have revealed a set of conserved transcription factors, as well as the Wnt signalling to be expressed in a similar manner among animals^1^. For instance, *caudal* expression is associated with the hindgut formation in members of ecdysozoans^9–11^, annelids^12,13^, cephalochordates^14^, molluscs^15^, echinoderms^16^, phoronids^17^ and vertebrates^18^. On the other hand, *brachyury* can be expressed in both the mouth and the hindgut (for example, in echinoderms, annelids and molluscs^19–21^), or only the mouth (for example, in chaetognaths^22^), or only the hindgut (for example, in ecdysozoans, phoronids and most chordates^17,23,24^). Finally, Wnt ligands and receptors are expressed in the hindgut of several animals, often interconnected with *brachyury* and *caudal*^25–35^. This common molecular profile led to the agreement that the hindgut of protostomes and deuterostomes (nephrozoans) share a common ancestry^11,19^.

Xenacoelomorpha (*Xenoturbella* + Acoelomorpha (Nemertodermatida + Acoela)) form the probable sister clade to the remaining bilaterians (Nephrozoa)^36–42^, although other positions have been suggested as well^43–45^. Like cnidarians, xenacoelomorphs possess a blind gut with only one opening, the mouth, which corresponds to the bilaterian mouth^2^ (Fig. 1). Their important phylogenetic position therefore holds the key to understanding the evolutionary transition from a blind, sac-like gut to a through gut in bilaterians (Fig. 1a). Interestingly, studies on the molecular patterning of the digestive system in two acoel species (*Convolutriloba longifissura* and *Symsagittifera roscoffensis*) showed that *caudal* and *brachyury*^1^ are demarcating the region around the male genital opening (gonopore), through which the sperm gets released to the exterior^2,46^. A putative evolutionary relationship of the acoel male gonopore and the bilaterian hindgut/anal opening was therefore postulated^2^, but not yet thoroughly investigated. Gonopores sensu stricto do not exist in non-bilaterian animals, thus gamete deposition takes place from different openings of the body; such as the mouth or transient openings (for example, Cnidaria)^47^ and the oscula (Porifera)^48^ (Fig. 1b). An exception is the Ctenophora, where gonopore-like structures are located along the comb rows^49^; however, given the distant phylogenetic position of ctenophores^50^, these structures do not seem to be homologous with the bilaterian gonopores (Fig. 1b). Therefore, Xenacoelomorpha—as the most likely sister taxon to the remaining Bilateria—is important in understanding whether the presence of gonopores is connected to the emergence of a through gut with an anus in the lineage of Bilateria (Fig. 1a). To test the hypothesis of the homology of the xenacoelomorph gonopore and bilaterian anus, we investigated more species from the xenacoelomorph clade and revealed the presence of the male gonopore and its relation to the expression of several conserved bilaterian foregut-, midgut- and hindgut-related genes^1,26,51^.

## Results

### *Xenoturbella bocki* possesses a male gonopore

**Fig. 2:**
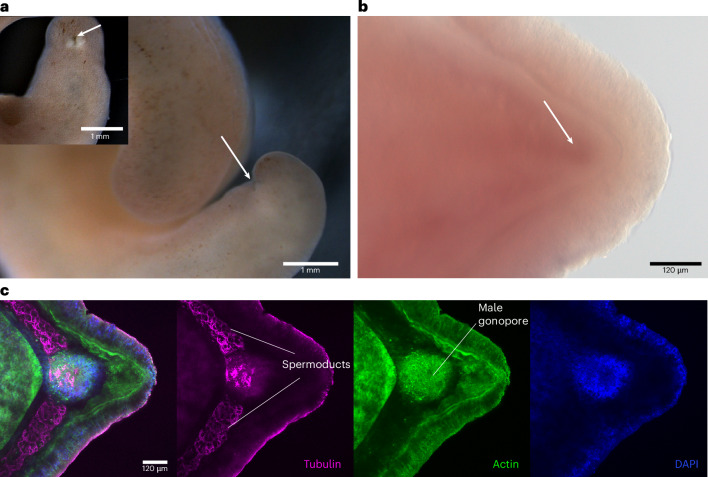
The male gonopore of *X. bocki.* **a**, Live image of an adult *X. bocki* and indication of a posterior male gonopore (white arrow). **b**, Differential interference contrast microscopy (DIC) image of the posterior part of an *X. bocki* adult and indication of a posterior male gonopore (white arrow). **c**, Immunohistochemistry of anti-tubulin (magenta), anti-actin (green) and nuclei (blue) of the posterior part of a *X. bocki* adult. Anti-tubulin is labelling the sperm. Every fluorescent image is a full projection of merged confocal stacks. Anterior is to the left.

The emergence of male gonopores probably occurred early in animal evolution, since spermatozoa are always released to the environment or transferred to a partner through a spermoduct and a gonopore^52^. In contrast, female gonopores are often missing in several animal lineages (for example, Catenulida and Gnathostomulida), and oocytes are released either through the mouth openings or body ruptures^53–55^. Similarly, in the hermaphroditic acoelomorphs the male gonopore has been described as part of the ground pattern, since it is present in all acoel and nemertodermatid species investigated so far^56^. In contrast, a female gonopore evolved within the lineage of Acoela and perhaps even more than once^56^. Although the last common ancestor of Acoelomorpha probably possessed only a male gonopore^57^, previous studies on individuals of their sister group, *Xenoturbella*, do not describe a male gonopore^58,59^. We revisited this subject by investigating the species *Xenoturbella bocki*. A single pore with bilaterally arranged white spots was observed posterior-ventrally of the living adult animal, resembling a male gonopore (Fig. 2a,b). Careful examination using immunofluorescence labelling confirmed the presence of two bilateral spermoducts and a central male gonopore at the posterior end of *X. bocki* (Fig. 2c). Why the prominent gonopore of *X. bocki* has not been described previously remains unclear. Since our specimen was collected during the winter (December), we could perhaps witness a seasonal appearance of the male gonopore. In line with previous work, we could not detect a female gonopore. Therefore, the presence of a male gonopore in *X. bocki* reflects the condition found in nemertodermatids and acoels. These results suggest that the last common ancestor of Xenacoelomorpha was characterized by a posterior male gonopore and lacked female reproductive openings.

### Bilaterian hindgut markers are expressed around the male gonopore of acoels

We first identified orthologous sequences of the foregut/midgut markers *goosecoid (gsc)*, *foxa*, *gata456*, *ptfa1* and *hnf4*, the hindgut markers *brachyury*, *caudal*, *even skipped (evx)* and *nk2.1*, as well as members of the Wnt signal pathway^1,26,51^ in the available transcriptomes or/and genomes of the acoels *Isodiametra pulchra*, *Convolutriloba macropyga*, *Hofstenia miamia* and the nemertodermatid *Meara stichopi* (Supplementary Figs. 1–15). We detected some gene duplications within the Acoela for *foxa*, *gata456*, *nk2.1* and *frizzled9/10* (Supplementary Figs. 10–12 and 15). Moreover, all acoel Wnt genes grouped in one clade with notable longer branch lengths and no clear orthology to other metazoan Wnts, suggesting that they are fast-evolving sequences, which do not allow their orthologization (Supplementary Figs. 1, 2 and4–6). On the contrary, clear orthologues of some nemertodermatid Wnts to other metazoans were found (Wnt1, Wnt3, Wnt5 and Wnt11 for *M. stichopi* and Wnt1, Wnt5, Wnt11 and Wnt16 for *Nemertoderma westbladi*) (Supplementary Figs. 1–3), as it was for the xenoturbellids *X. bocki* and *Xenoturbella profunda* (Wnt4, Wnt5, Wnt6, Wnt7, Wnt8 and Wnt10 for both species plus Wnt2, Wnt3, Wnt11 and Wnt16 for *X. bocki*) (Supplementary Figs. 1 and 2). With the exception of Wnt1, our results are similar to the previous Wnt gene characterization performed in *X. bocki*^60^.

**Fig. 3:**
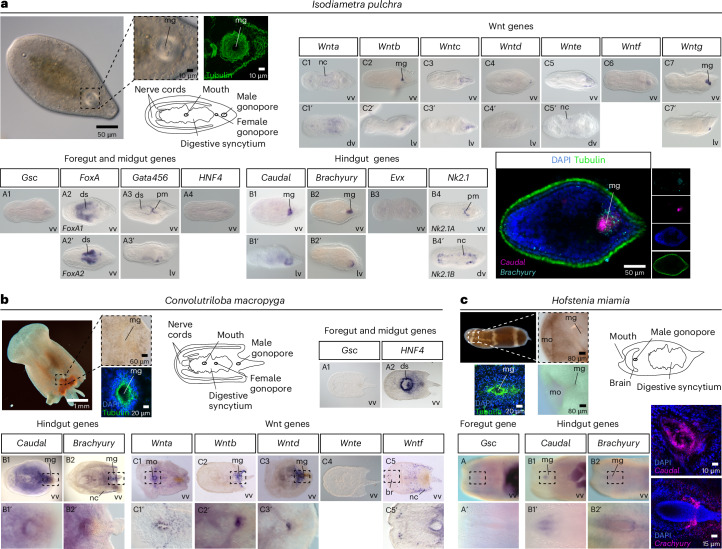
Gene expression of bilaterian foregut/midgut and hindgut markers in different acoel species. **a**–**c**, WMISH of the foregut and midgut markers *gsc*, *foxA*, *gata456* and *hnf4*, the hindgut markers *caudal* and *brachyury* and members of the Wnt family in *I. pulchra* (**a**), *C. macropyga* (**b**) and *H. miamia* (**c**). In *I. pulchra* the male gonopore is depicted with immunofluorescence using anti-actin (green). In fluorescent WMISH, *caudal* and *brachyury* expression is in magenta and nuclei are in blue. In double fluorescent WMISH, *brachyury* expression is in cyan, *caudal* is in magenta, tubulin is in green and nuclei are in blue. Drawings are not to scale. Anterior is to the left. ds, digestive syncytium; dv, dorsal view; lv, lateral view; nc, neural cells; pm, parenchymal muscles; vv, ventral view.

**Extended Data Fig. 1:**
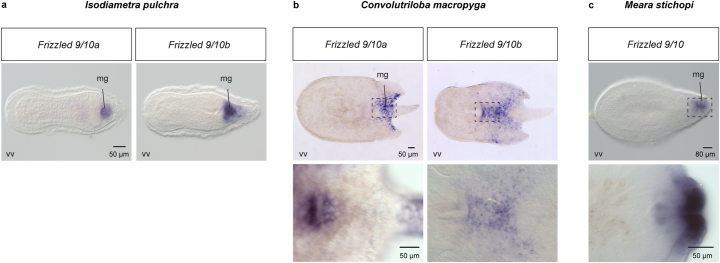
Frizzled9/10 gene expression in acoelomorphs. WMISH of *frizzled9/10* genes in (**a**) *Isodiametra pulchra*, (**b**) *Meara stichopi* and (c) *Convolutriloba macropyga*. Anterior to the left. mg, male gonopore; vv, ventral view.

**Extended Data Fig. 2:**
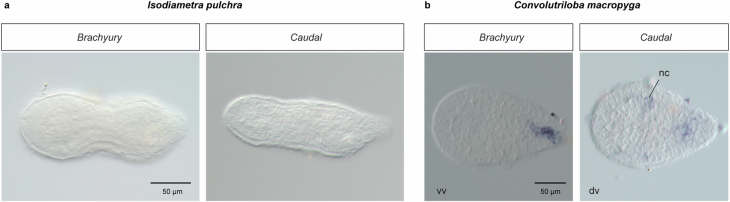
Caudal and brachyury gene expression in acoels. WMISH of *brachyury* and *caudal* in the juveniles of the acoels (**a**) *Isodiametra pulchra* and (**b**) *Convolutriloba macropyga*. Anterior to the left. dv, dorsal view; nc, neural cells; vv, ventral view.

We then examined the expression of these genes by whole-mount in situ hybridization (WMISH) in adult, sexually matured animals, where the male gonopore was already formed. In the acoel *I. pulchra*, the male gonopore is situated at the posterior end of the animal, just posterior to the female gonopore^61^ (Fig. 3a). The two paralogues of *foxA*^62^ were expressed along the digestive syncytium (Fig. 3a (A2)), while transcripts of *gata456*^62^ were labelling the posterior digestive syncytium and muscles (Fig. 3a (A3)). Interestingly, the hindgut markers *caudal* (Fig. 3a (B1)) and *brachyury* (Fig. 3a (B2)), but also two members of the Wnt family (*wntb* and *wntg*) (Fig. 3a (C2,C7)) and their receptors *frizzled9/10a* and *frizzled9/10b* (Extended Data Fig. 1a) were all expressed around the male gonopore. Other members of the Wnt family were expressed in neuronal cells and cells of the posterior tip (Fig. 3a (C1,C3–6)). Finally, the two paralogues *nk2.1a* and *nk2.1b* were expressed in the parenchymal muscles and neuronal cells, respectively (Fig. 3a (B4)). Double WMISH experiments showed that *brachyury* and *caudal* were co-expressed around the male gonopore (Fig. 3a). However, the early juveniles of *I. pulchra* that do not have a gonopore yet were devoid of *brachyury* and *caudal* expression (Extended Data Fig. 2a), suggesting that the expression implies an active role during the male gonopore formation.

In the acoel *C. macropyga*, the male gonopore is located at the posterior region of the animal, posteriorly to the female gonopore^63^ (Fig. 3b). Transcripts of *hnf4* were localized in the digestive syncytium (Fig. 3b (A2)). Again, the hindgut markers *brachyury* and *caudal* (Fig. 3b (B1,B2))*, wntb* and *wntd* (Fig. 3b (C2,C3)), as well as *frizzled9/10a* (Extended Data Fig. 1b) were expressed around the male gonopore. Other members of the Wnt family were expressed in individual neuronal cells and the mouth (Fig. 3b (C1,C5)). In immature *C. macropyga* without gonopores, *brachyury* was expressed posteriorly and transcripts of *caudal* were solely localized in the developing nervous system (Extended Data Fig. 2b). The expression of *brachyury* and *caudal*in*C. macropyga* embryos resembles the previously reported expression in a different *Convolutriloba* species^2^. However, the expression becomes prominent around the male gonopore at the adult stage, suggesting that these genes have a distinct gonopore-specific role. Interestingly, none of the investigated genes was expressed in the female gonopore region in both species. This observation reinforces the plesiomorphic condition of the male gonopore within Acoela and further suggests that the molecular patterning of the two gonopores is different.

### The expression of *brachyury*, *caudal* and components of the Wnt pathway is gonopore specific

**Extended Data Fig. 3:**
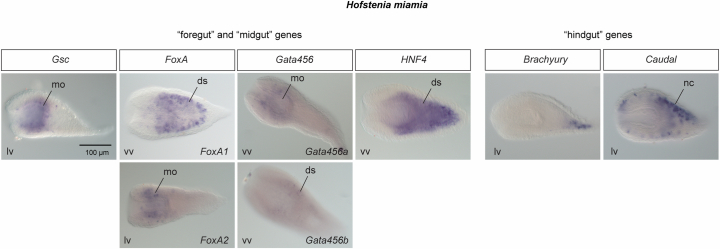
Gene expression of the bilaterian foregut/ midgut markers and hindgut markers in the acoel *Hofstenia miamia*. WMISH of bilaterian foregut/ midgut markers and hindgut markers in the juveniles of the acoel *Hofstenia miamia*. WMISH of the foregut and midgut markers *gsc*, *foxA, gata456* and *hnf4* and the hindgut markers *caudal* and *brachyury* in *Hofstenia miamia* juveniles. Anterior to the left. ds, digestive syncytium; lv, lateral view; mo, mouth; nc, neural cells; vv, ventral view.

Since Brachyury, Caudal and the Wnt pathways are involved in the posterior patterning and growth in several animals^9,27,28,31,64–69^, we aimed to discriminate whether the witnessed gene expression is gonopore specific or related to the overall posterior patterning of the animal. Therefore, we investigated the acoel species *H. miamia* where the position of the male gonopore is located anterior in the body, just posterior to the anterior mouth^70^ (Fig. 3c). Remarkably, both hindgut markers *brachyury* and *caudal* were expressed around the anterior male gonopore in *H. miamia*, separated from the posterior tip of the animal (Fig. 3c (B1,B2)). The expression profiles of the Wnt genes (*wnt1*) and the Frizzled receptors (*fz-1)* were reported in a previous publication in the region of the male gonopore^42^. In fact, *wnt1* is an orthologue of *wntg* of the acoel *I. pulchra* (Supplementary Figs. 1 and2), which also demarcates the region around the male gonopore (Fig. 3a (C7)). However, in the early juveniles where the gonopore is not yet formed, *brachyury* was expressed posteriorly and *caudal* was only seen in the developing nervous system, suggesting that their expression around the male gonopore is not a remnant of an earlier embryonic patterning but is gonopore specific (Extended Data Fig. 3).

These findings show that in all three investigated acoel species, the expression of the hindgut markers *caudal* and *brachyury*, and members of the Wnt family and Frizzled receptors are consistently expressed around the male gonopore, suggesting that the molecular patterning of the male gonopore is conserved among acoels and bears strong similarities to the bilaterian hindgut.

### Bilaterian hindgut markers are expressed around the male gonopore of nemertodermatids

**Fig. 4:**
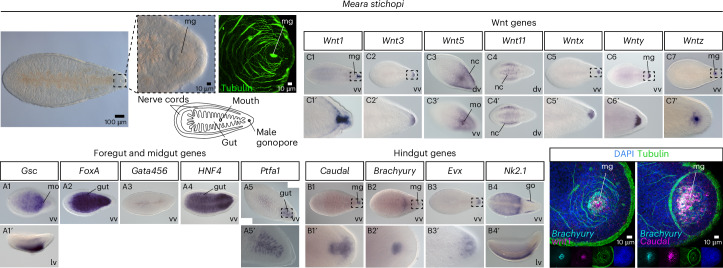
Gene expression of bilaterian foregut/midgut and hindgut markers in nemertodermatids. WMISH of the foregut and midgut markers *gsc*, *foxA*, *gata456*, *ptf1* and *hnf4*, the hindgut markers *caudal* and *brachyury* and members of the Wnt family in *M. stichopi*. The male gonopore is depicted with immunofluorescence using anti-tubulin (green). In double fluorescent WMISH, *brachyury* expression is in cyan, *caudal* and *wnt1* expression is in magenta, tubulin is in green and nuclei are in blue. Drawings are not to scale. Anterior is to the left. go, female gonads.

**Extended Data Fig. 4:**
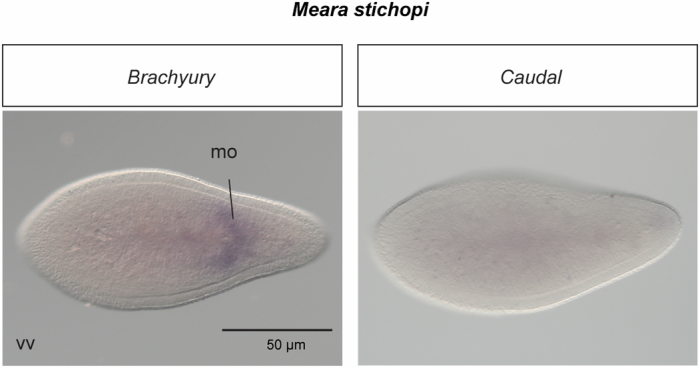
*Caudal* and *brachyury* gene expression in nemertodermatids. WMISH of *brachyury* and *caudal* in the juveniles of the nemertodermatid *Meara stichopi*. Anterior to the left. mo, mouth; vv, ventral view.

To understand whether this molecular conservation extends to the lineage of nemertodermatids, we investigated the expression of the aforementioned genes in the nemertodermatid *M. stichopi*. Nemertodermatids are the sister group to acoels, and they possess an epithelial gut versus a digestive syncytium that is an evolutionarily derived character of acoels^71^. The male gonopore is placed at the posterior end, while a female gonopore is absent^72^ (Fig. 4). Marker *gsc* was expressed in the mouth (Fig. 4 (A1)) and transcripts of *foxa* (Fig. 4 (A2)) and *hnf4* (Fig. 4 (A4)) were localized in the entire gut. Marker *ptfa1* was expressed only in the posterior part of the gut (Fig. 4 (A5)). The hindgut markers *caudal* (Fig. 4 (B1)) and *brachyury* (Fig. 4 (B2)) were expressed around the male gonopore, while *evx* was expressed at the posterior tip (Fig. 4 (B3)) and *nk2.1* showed a ventral expression (Fig. 4 (B4)). Moreover, three members of the Wnt family (*wnt1*, *wnty* and *wntz*) (Fig. 4 (C1,C6,C7)) and the receptor *frizzled9/10* (Extended Data Fig. 1c) were expressed around the male gonopore. Other members of the Wnt family were marking neuronal cells and cells of the posterior tip (Fig. 4 (C2–C5)). Transcripts of *nk2.1* were additionally demarcating the female gonadal region (Fig. 4 (B4)) and *brachyury* was also expressed in the mouth (Fig. 4 (B2)). Double WMISH analysis confirmed that *brachyury* and *caudal* were co-expressed around the male gonopore and the same was true for *brachyury* and *wnt1* (Fig. 4). Finally, as in the acoels, the immature juveniles of the nemertodermatid *M. stichopi* that do not possess a gonopore yet, *brachyury* expression was restricted in the future mouth region—the mouth forms later in nemertodermatids^73^—and *caudal* was absent (Extended Data Fig. 4).

In general, these findings demonstrate that in all investigated acoel and nemertodermatid species, the bilaterian hindgut markers are specifically expressed in the male gonopore region during its formation. However, the same is not true for the foregut/midgut markers, which are only expressed in regions associated with the digestive system of acoelomorphs. Therefore, these results support the homology of the male gonopore and the hindgut^2^.

## Discussion

### A distinct, later role of *brachyury*, *caudal* and *Wnt* cascade is used for the formation of acoelomorph gonopore

*Caudal* and *brachyury* are involved in posterior patterning in diverse species^9,27,28,31,64–69^, often associated with the Wnt pathway^67^. Although in xenacoelomorphs such function is not tested functionally, it has been shown that the Wnt pathway is involved in posterior regeneration in the acoel *H. miamia*^74^, which suggests a Wnt-dependent, evolutionarily conserved mechanism for establishing the identity of posterior tissues. Indeed some *wnt* genes are expressed in the posterior tip of the acoels *I. pulchra* (Fig. 3a (C4,C5))*, C. macropyga* (Fig. 3a (C2)) and the nemertodermatid *M. stichopi* (Fig. 4 (C2,C5)), so their participation in the establishment of acoelomorph axial identities is likely. *Brachyury* is expressed transiently at the posterior end of all three investigated acoel species only at earlier stages (Extended Data Figs. 2 and 3). The fact that the early expression of *brachyury* in the acoel *H. miamia* is also posteriorly and not anteriorly, where the gonopore forms (Extended Data Fig. 3), suggests that it is independent of gonopore formation. Therefore, in acoelomorphs *brachyury* probably has two separate roles: an early role in establishing posterior identities and a later role in gonopore patterning. However, *caudal* is never expressed posteriorly (Extended Data Figs. 2 and 4), thus suggesting that this gene does not have any role in posterior patterning and it is only used for firstly patterning neuronal tissues and later on for gonopore formation in acoelomorphs.

In adult acoelomorphs, *caudal* and *brachyury* expression is demarcating solely the male gonopore, in combination with some members of the *wnt* and *frizzled* family. The witnessed expression of *caudal*, *brachyury* and *wnt* genes in the acoel *H. miamia* that possesses an anterior and not a posterior gonopore, further support their specific relation to gonopore formation and not in posterior patterning. Finally, the fact that the combinatory expression of the *caudal*/*brachyury*/*wnt* toolkit is demarcating solely the male gonopore and not other openings in the acoelomorph body, such as the female gonopore and the mouth, demonstrates that it is not part of a general programme for making ‘openings’, but is associated exclusively to the formation of the male gonopore.

### An ancestral molecular patterning is used for hindgut and male gonopore formation

**Fig. 5:**
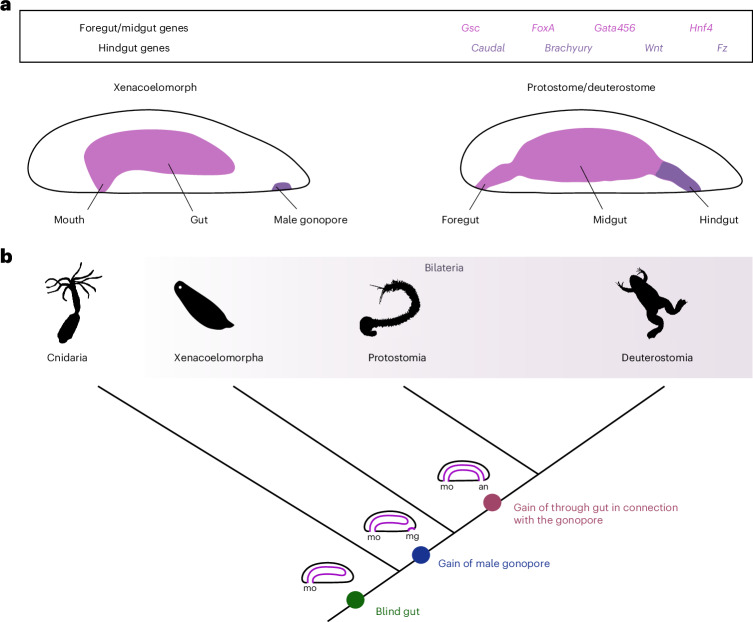
Homology of the xenacoelomorph male gonopore and the bilaterian anus. **a**, Summary of bilaterian foregut/midgut- and hindgut-related gene expression in Nephrozoa (protostomes + deuterostomes) and Xenacoelomorpha. The *brachyury*, *caudal* and *wnt* ligands and receptors are expressed in the male gonopore of acoelomorphs and the hindgut of nephrozoans, suggesting their homology. The hindgut markers were initially expressed in the gonopore and afterwards they were assigned in patterning the hindgut/anal opening. **b**, The last common bilaterian ancestor possessed a blind gut and a gonopore, and gained a hindgut/anal opening in connection with the gonopore after the Xenacoelomopha/Nephrozoa split. Credit: Silhouettes from phylopic.org under a Creative Commons license: *Diopatra cuprea* (CC BY 4.0), *Xenopus laevis* (CC BY 3.0), *Nematostella vectensis* and *Dugesia* *japonica* (CC0 1.0).

The hindgut of most bilaterians forms by the expression of *brachyury* and *caudal* transcriptional regulators, as well as the Wnt signalling pathway^1^. Xenacoelomorphs, the most likely sister group to all remaining bilaterians^36–42^, lack a through gut so they do not use these bilaterian hindgut-related genes and the Wnt signalling cascade for patterning their gut. Instead these genes are used for patterning their male gonopore in the late juvenile/adult stages (Fig. 5a). In non-bilaterians, which also lack a through gut (except perhaps for the Ctenophora, where the anal pores with anus-like characteristics has been described^75^, but due to the distant phylogenetic position of this taxon^50^ it seems to be a derived feature), the expression of these genes is variable in later stages of development. For instance, *brachyury* seems to be expressed in species-specific differentiated tissues (for example, the tentacular buds of the Ctenophora^76^, the mesenteries (in *Nematostella*) or the hypostome (in *Hydra*) of the Cnidaria^77,78^, the choanocytes of the Porifera^79^ and isolated cells located at the edge of the organism in the Placozoa^80^). In contrast, *caudal* gene appears to be missing from the Ctenophora, Placozoa and Porifera^81^. In cnidarians a *caudal*/*xlox* orthologue is expressed differently among species (for example, in the ventral pair of mesenteries of *Nematostella*^82^ and in both the oral and aboral pole in *Clytia*^83^). Finally, the Wnt signalling in non-bilaterians is mainly necessary for the formation of a primary, aboral–oral and/or anterior–posterior body axis, a function also witnessed in the early developmental stages of bilaterian species^84^, often achieved via an ancestral feedback loop with *brachyury*^85^. The absence of a clear *caudal* orthologue, the variability of the late *brachyury* expression and the main role of Wnt pathway as a polarization, symmetry-brake signal in non-bilaterian animals imply that the assignment of these genes in patterning the posterior digestive system (hindgut) and the xenacoelomorph gonopore probably occurred after the Bilateria/Cnidaria split. Since the male gonopore of most xenacoelomorphs forms by involution of the posterior ectoderm, a similar process that occurs during the anus formation of several metazoans^3,5^, we conclude that the same ancestral posterior ectodermal molecular patterning is used for both the hindgut/anal canal and the male gonopore formation. These findings are in line with the theory supporting that the through gut evolved at the lineage of nephrozoans and the bilaterian mouth corresponds to the cnidarian/xenacoelomorph mouth^2^, and further suggest that the male gonopore of xenacoelomorphs and the bilaterian anus are homologous structures.

### The bilaterian anus evolved from a male gonopore

Although our data suggest the homology of the xenacoelomorph male gonopore with the bilaterian anus, the direction of evolutionary transition between a gonopore and a hindgut with an anus can be interpreted in different ways, depending on (1) the phylogenetic position of Xenacoelomorpha as sister to the remaining Bilateria^36–42^ or sister to Ambulacraria^43–45^ and (2) whether the absence of an anal opening is plesiomorphic for the Bilateria. The main difference is whether the last common ancestor possessed a gonopore and a blind gut without an anal opening or a through gut with a cloaca combining both genital and digestive functions. Morphologists have developed different hypotheses in the past^86–88^, where Platyhelminthes and Acoelomorpha were selected as examples of transition states because of their variable digestive and genital system architectures. The presence of cloaca within animals as well as the gonopore–oral fusion witnessed in species of Platyhelminthes (Fig. 1b), suggests that a connection between the digestive and the reproductive system is either easy to evolve convergently or shares a common ancestry.

Revisiting the subject adding molecular data and using better phylogenies we can now propose that the last common ancestor probably possessed a blind digestive tract with a mid-ventrally placed single opening which functioned as a mouth expressing *gsc*, *foxa, gata456* and *hfn4*, and a posterior male gonopore expressing *caudal*, *brachyury* formed under a Wnt signal, similar to what is found in acoels and nemertodermatids. This (ectodermal) male gonopore region was later connected with the posterior domain of the endodermal blind digestive tract resulting in the acquisition of a hindgut and the subsequent formation of an anus, perhaps in the form of a cloaca (Fig. 5b), similarly to the old theory of ref. ^88^, in which the hindgut evolved from the genital system in the form of a cloaca. These ‘gonopore’ genes were then recruited in patterning the hindgut, suggesting that the anus evolved in connection with the male gonopore.

The alternative but less parsimonious scenario would be that the last common ancestor possessed a through gut with an anus expressing hindgut-related genes, and the hindgut with the anal opening was secondarily lost in the lineage of xenacoelomorphs. The expression of ‘hindgut’ markers around the male gonopore of acoelomorphs should be then interpreted as a secondary recruitment of these genes to the posterior ectodermal/endodermal boundary, where the male gonopore forms. However, although an ancestral condition of a through gut cannot be excluded, a plesiomorphic blind gut that gained later an anal opening in connection with the gonopore seems more likely, especially in the scenario where the Xenacoelomorpha are sister to the remaining Bilateria (Fig. 5b). In fact, these genes are never expressed in association with the digestive system in acoelomorphs indicating that the posterior portion of the gut had not evolved yet in this lineage. Our findings provide strong molecular evidence for the homology of the male gonopore of the Xenacoelomorpha and the anus of the Bilateria.

## Methods

### Animals

Adult specimens of *I. pulchra* Smith and Bush, 1991, *M. stichopi* Westblad, 1949, *H. miamia* Correa, 1960 and *C. macropyga*, Shannon and Achatz, 2007 were kept and handled as previously described^63,89,90^. *X. bocki*, Westblad, 1949 were obtained from Gullmarsfjord, Sweden.

### Gene cloning and orthology assignment

Putative orthologous sequences of genes of interest were identified by tBLASTx search against the transcriptome (SRR2681926) of *I. pulchra*, the transcriptome (SRR2681155) and draft genome of *M. stichopi*, the transcriptome (SRX1343815) of *C. macropyga* and the transcriptome (PRJNA241459) and genome (GCA_004352715) of *H. miamia*. Gene orthology of genes of interest identified by tBLASTx was tested by reciprocal BLAST against National Center for Biotechnology Information Genbank and followed by phylogenetic analyses. Protein sequences were aligned using MAFFT (v.7.487) with the L-INS-i strategy (--amino --localpair --maxiterate 1000) to account for local homology and maximize alignment accuracy. Poorly aligned regions were trimmed using trimAl (v.1.4.rev15) with either automated trimming (-automated1) or a gap threshold of 0.5 (-gt 0.5), which removes columns containing gaps in more than 50% of sequences. The alignments are provided in ref. ^91^. For the Wnt tree, a trimming threshold (-gt 0.1), removing columns with gaps in more than 10% of the sequences was used. The best-fitting evolutionary model for each gene tree was selected using ModelTest-NG (v.0.1.7), and maximum likelihood phylogenies were reconstructed using RAxML-NG (v.1.2.1). To estimate node support, we performed bootstrap analysis using RAxML-NG with the autoMRE stopping criterion, allowing up to 5,000 replicates (--bs-trees autoMRE (5000)). This approach adaptively determines the sufficient number of bootstrap replicates by monitoring the stability of the consensus tree. The evolutionary models used for each tree (automated trimming/gap threshold of 0.5) were: LG + G4/JTT + G4 (for *hnf4*), JTT + I + G4/VT + I + G4 (for *foxa* and *nk2*.1), LG + I + G4/JTT + I + G4 (for *brachyury*), LG + G4/VT + I + G4 (for *cdx* and *evx*), VT + I + G4/VT + I + G4 (for *gsc*), JTT + I + G4/JTT + I + G4 (for *gata456*), LG + I + G4/LG + I + G4 (for *frizzled*) and LG + G8 + F (for *wnt*). Fragments of the genes of interest were amplified from complementary DNA of each animal by polymerase chain reaction (PCR) using gene-specific primers. PCR products were purified and cloned into a pGEM-T Easy vector (Promega) according to the manufacturer’s instruction and the identity of inserts confirmed by sequencing.

### Whole-mount in situ hybridization

Embryos were manually collected, fixed and processed for colorimetric and double fluorescent in situ hybridization as described in refs. ^2,92^. Labelled antisense RNA probes were transcribed from linearized DNA using digoxigenin-11-UTP (Roche) or labelled with dinitrophenyl (Mirus) according to the manufacturer’s instructions. The experiments were repeated at least twice and led to a reproducible pattern. The expression patterns were consistent between the different samples.

### Whole-mount immunohistochemistry

Animals were collected manually, fixed in 4% paraformaldehyde in seawater for 60 min, washed three times in phosphate buffered saline (PBS) + 2% bovine serum albumin (BSA) + 0.1% Tween 20 and incubated in 4% sheep serum in PBT for 30 min. The animals were then incubated with commercially available primary antibodies (acetylated tubulin mouse monoclonal antibody, dilution 1:250 (Sigma-Aldrich) and actin mouse monoclonal antibody, dilution 1:400 (Seven Hills Bioreagents) overnight at 4 °C, washed five times in PBT and followed by incubation in 4% sheep serum in PBT for 30 min. Specimens were then incubated with a secondary antibody overnight at 4 °C followed by five washes in PTW (PBS + 0.1% Tween 20). Nuclei were stained with 4′,6-diamidino-2-phenylindole (DAPI) (Invitrogen). The results between the different samples and experiments were consistent.

### Documentation

Colorimetric WMISH specimens were imaged with a Zeiss AxioCam HRc mounted on a Zeiss Axioscope A1 equipped with Nomarski optics and processed through Photoshop CS6 (Adobe). Fluorescent-labelled specimens were analysed with a SP5 confocal laser microscope (Leica) and processed by the ImageJ software v.2.0.0-rc-42/1.50d (Wayne Rasband). Figure plates were arranged with Illustrator CS6 (Adobe).

### Reporting summary

Further information on research design is available in the Nature Portfolio Reporting Summary linked to this article.

## Supplementary information

Supplementary Information Supplementary Figs. 1–15: Phylogenetic analyses of Wnt, Brachyury, Caudal/Cdx, Evx, FoxA, Frizzled, Gata4/5/6, Gsc, Hnf4 and Nk2.1 sequences. Names of genes or proteins, if available, follow the name of organism(s). *I. pulchra*, *M. stichopi*, *H. miamia* and *C. macropyga* sequences are highlighted in purple, dark blue, orange and light green, respectively. Other xenacoelomorph sequences included in the analysis are taken from the transcriptomes of *X. bocki* (in magenta), *X. profunda* (in red), *N. westbladi* (in cyan), *Praesagittifera naikaiensis* (in yellow), *S. roscoffensis* (in grey), *Diopisthoporus longitubus* (in dark green) and *Ascoparia*sp. (in light blue). Supplementary Tables 1–4: List of primer sequences used in the study.

Reporting Summary

## Data Availability

All newly determined sequences have been deposited in GenBank under accession numbers PV710562–PV710580. The phylogenetic tree alignments are available via Zenodo at https://zenodo.org/records/15555485 (ref. ^91^). Primer sequences are available in Supplementary Tables 1–4.
