# Supplementary material for: Molecular evidence from xenacoelomorph gonopore formation supports homology with the bilaterian anus
Source: Nat Ecol Evol. 2025 Oct 24;9(11):2116–26. doi: 10.1038/s41559-025-02866-6 (PMC12592216; doi:10.1038/s41559-025-02866-6)
Supplement: Supplementary file 1 — Supplementary Figs. 1–15: Phylogenetic analyses of Wnt, Brachyury, Caudal/Cdx, Evx, FoxA, Frizzled, Gata4/5/6, Gsc, Hnf4 and Nk2.1 sequences. Names of genes or proteins, if available, follow the name of organism(s). I. pulchra, M. stichopi, H. miamia and C. macropyga sequences are highlighted in purple, dark blue, orange and light green, respectively. Other xenacoelomorph sequences included in the analysis are taken from the transcriptomes of X. bocki (in magenta), X. profunda (in red), N. westbladi (in cyan), Praesagittifera naikaiensis (in yellow), S. roscoffensis (in grey), Diopisthoporus longitubus (in dark green) and Ascopariasp. (in light blue). Supplementary Tables 1–4: List of primer sequences used in the study. [file 41559_2025_2866_MOESM1_ESM.pdf]

nature ecology & evolution

Supplementary information https://doi.org/10.1038/s41559-025-02866-6

Molecular evidence from xenacoelomorph gonopore formation supports homology with the bilaterian anus

In the format provided by the authors and unedited

Supplementary Figure 1

Phylogenetic analysis of several metazoan Wnt sequences

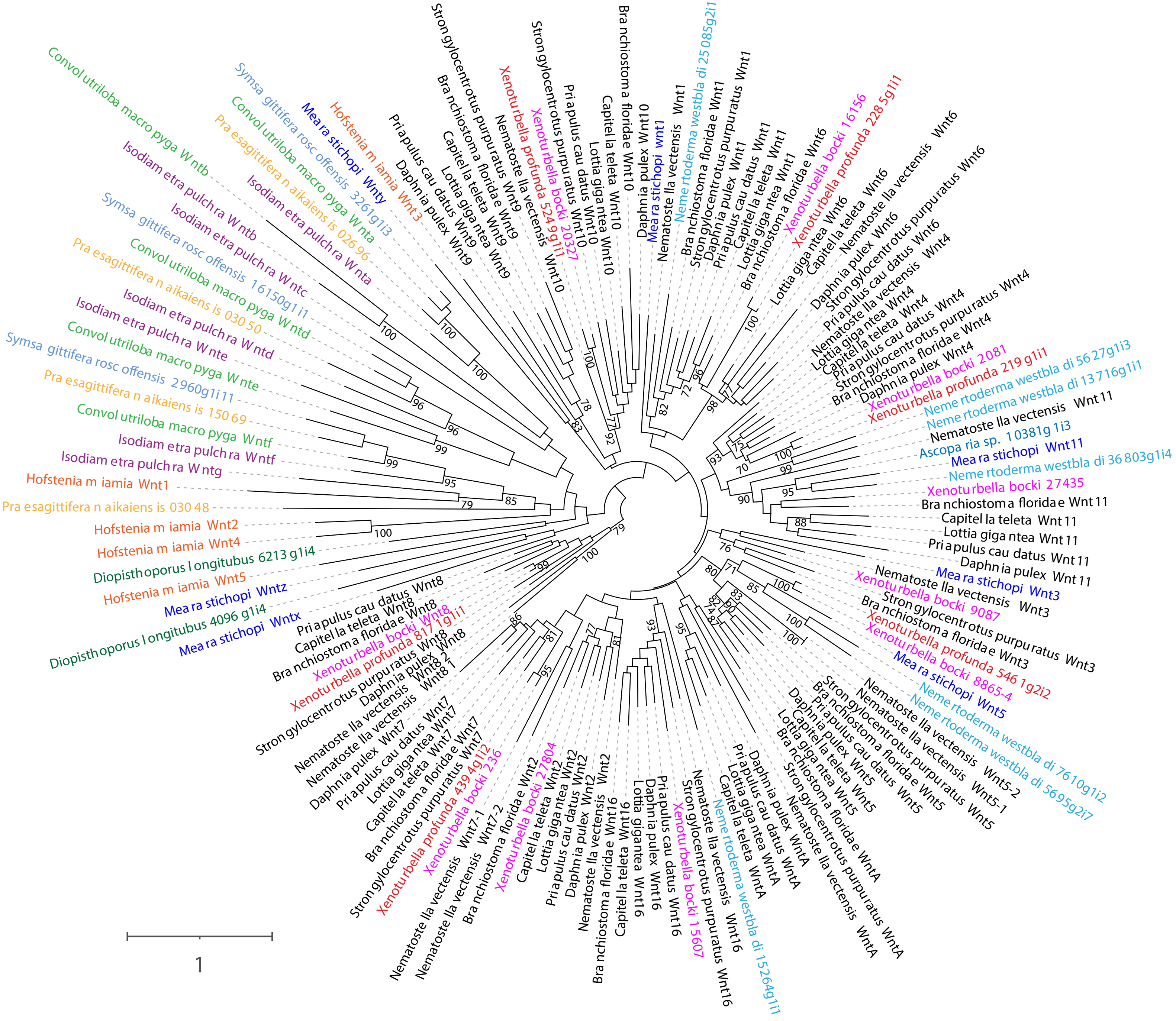

Suppementary Figure 2

Phylogenetic analysis of xenacoelomorph Wnt sequences

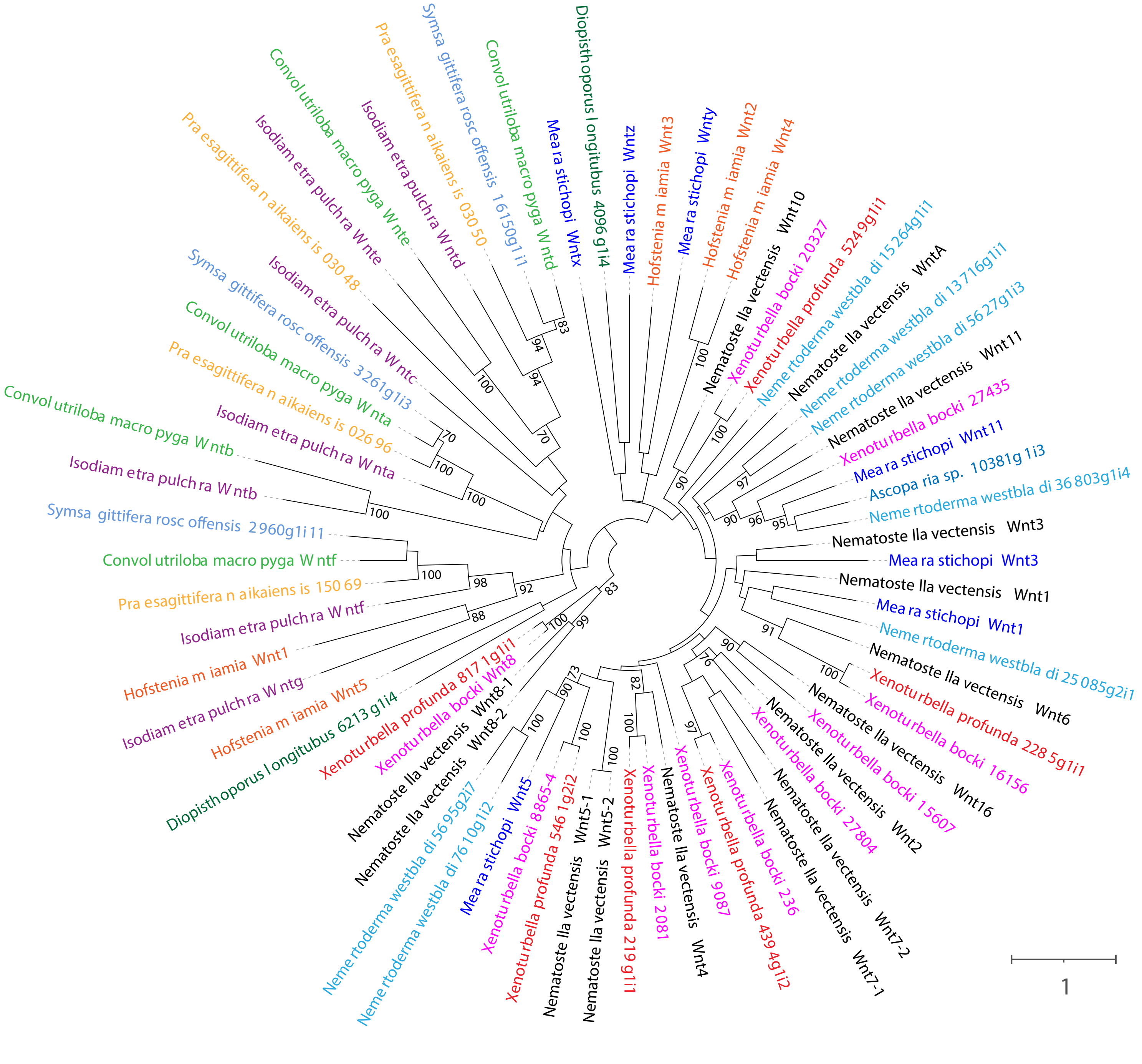

Supplementary Figure 3

Phylogenetic analysis of several metazoan and *Meara stichopi* Wnt sequences

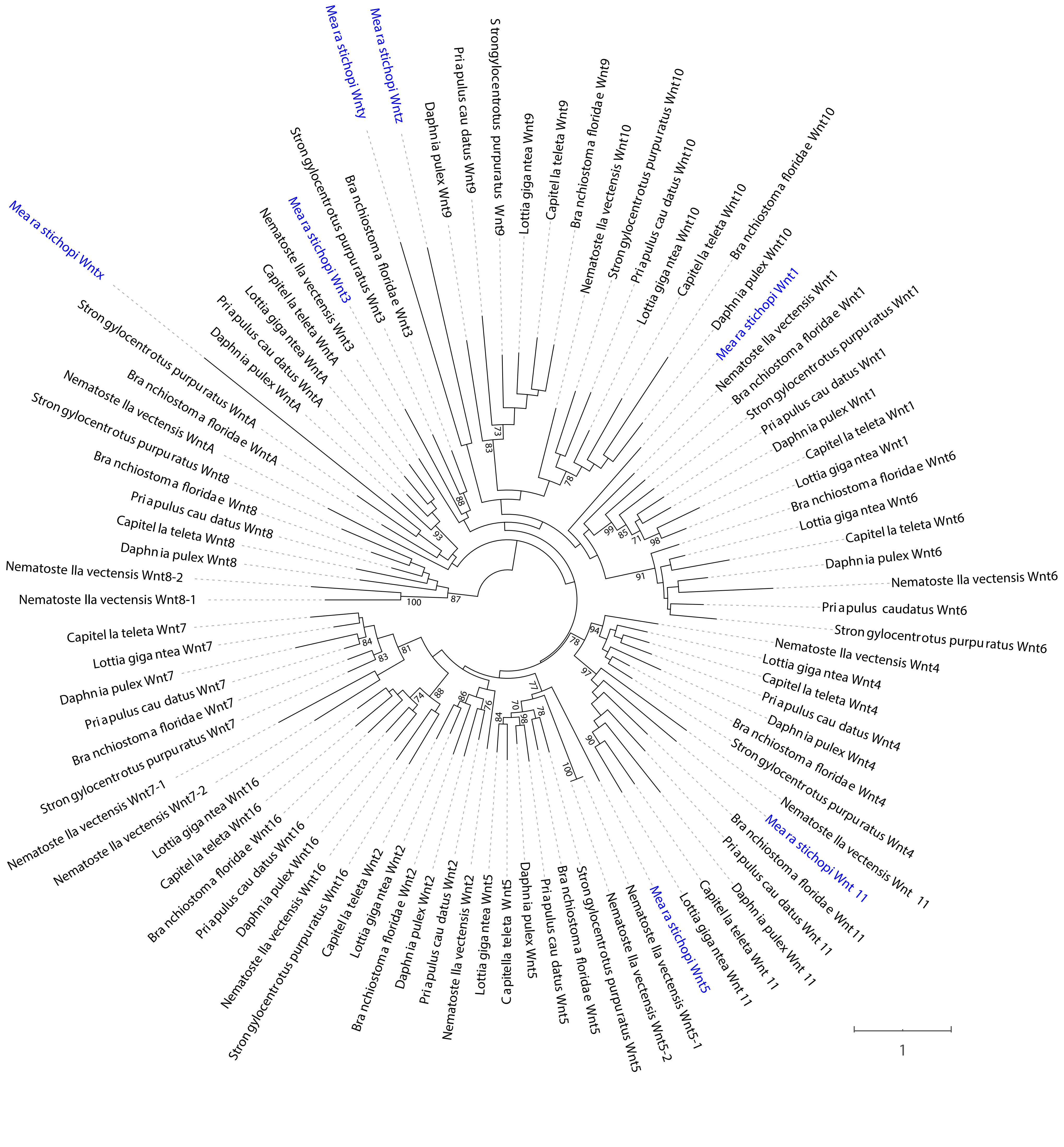

Supplementary Figure 4

Phylogenetic analysis of several metazoan and *Hofstenia miamia* Wnt sequences

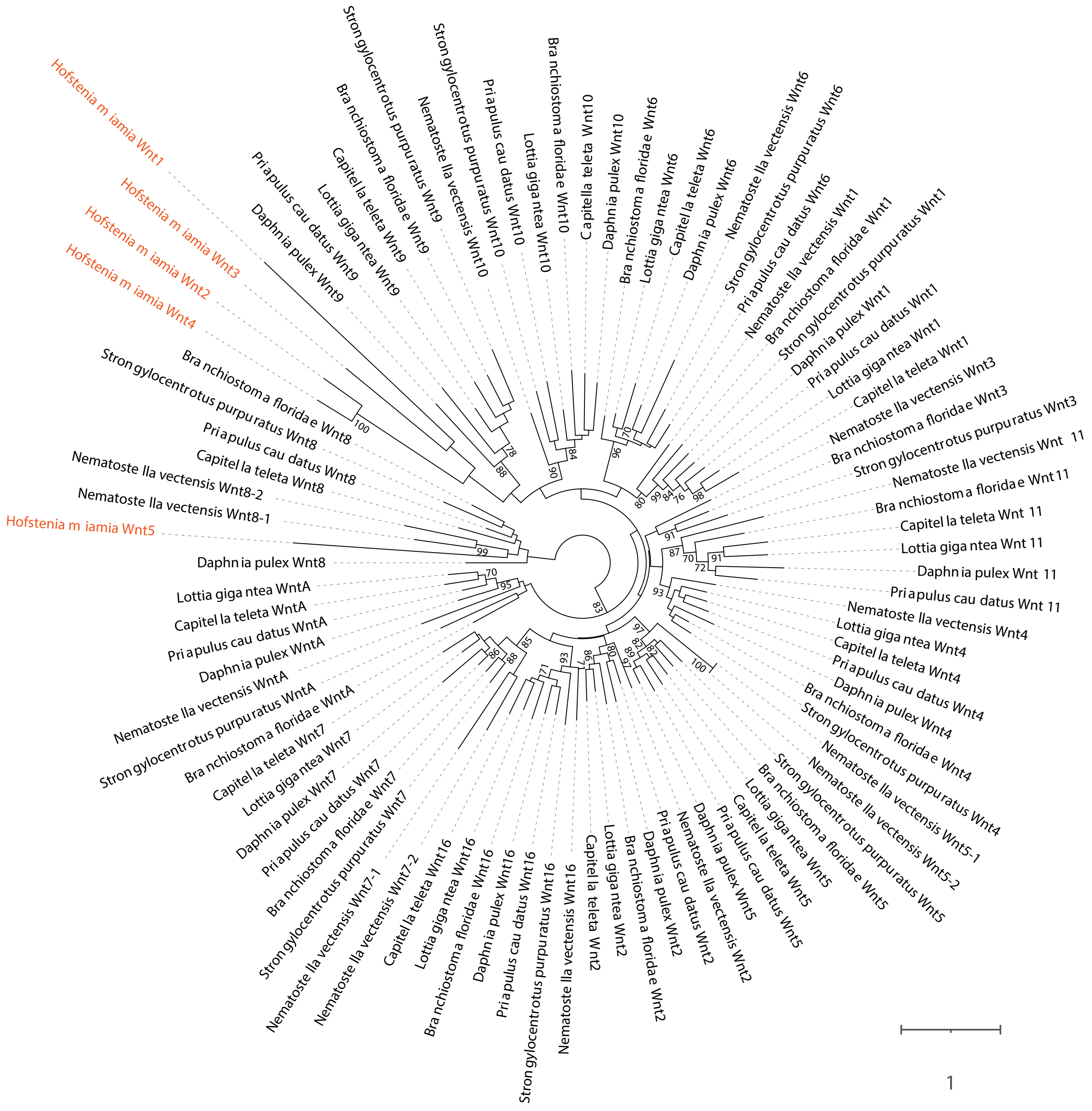

Supplementary Figure 5

Phylogenetic analysis of several metazoan and *Convolutriloba macropyga* Wnt sequences

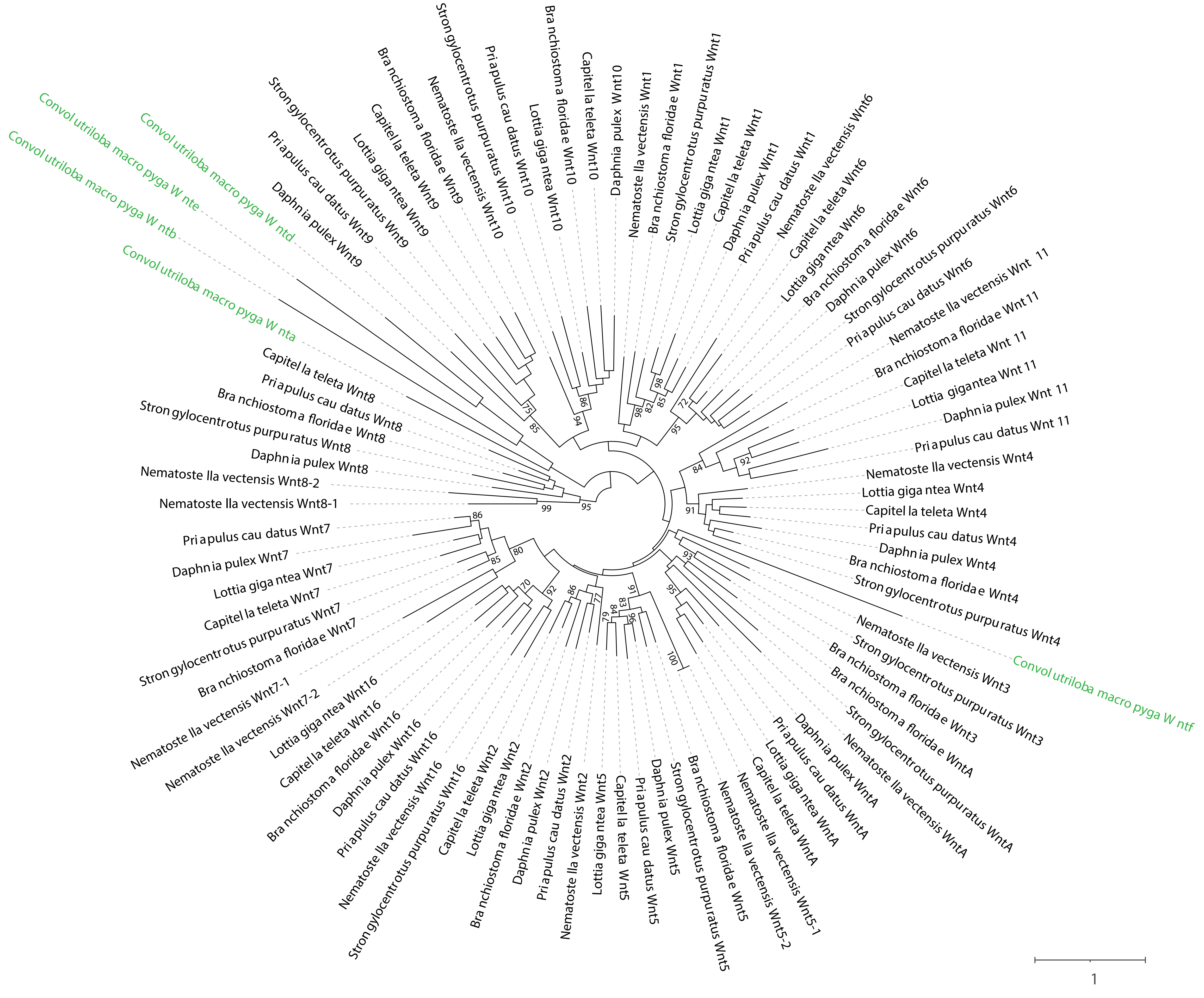

Supplementary Figure 6

Phylogenetic analysis of several metazoan and *Isodiametra pulchra* Wnt sequences

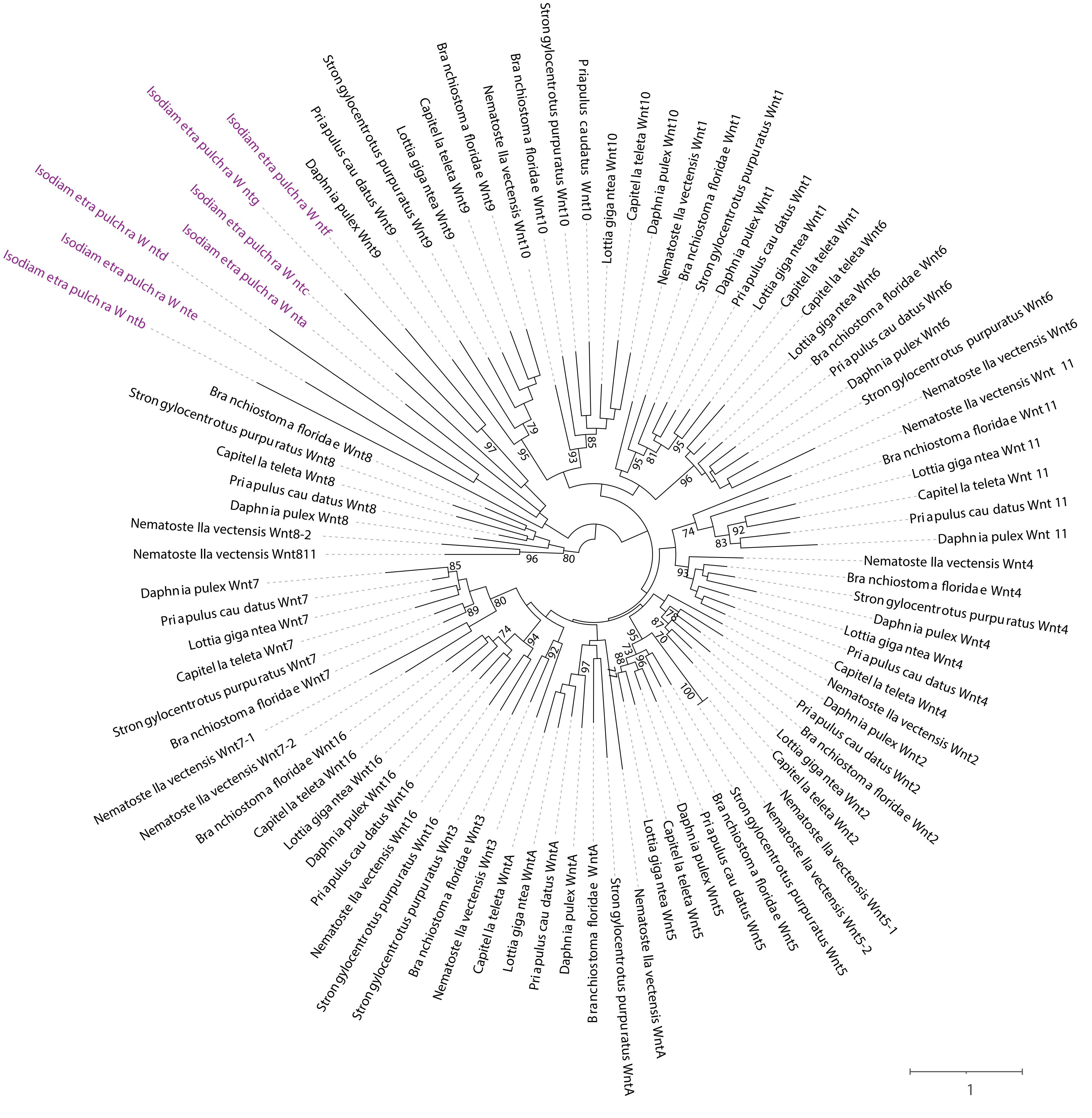

Supplementary Figure 7
Phylogenetic analysis of Brachyury sequences

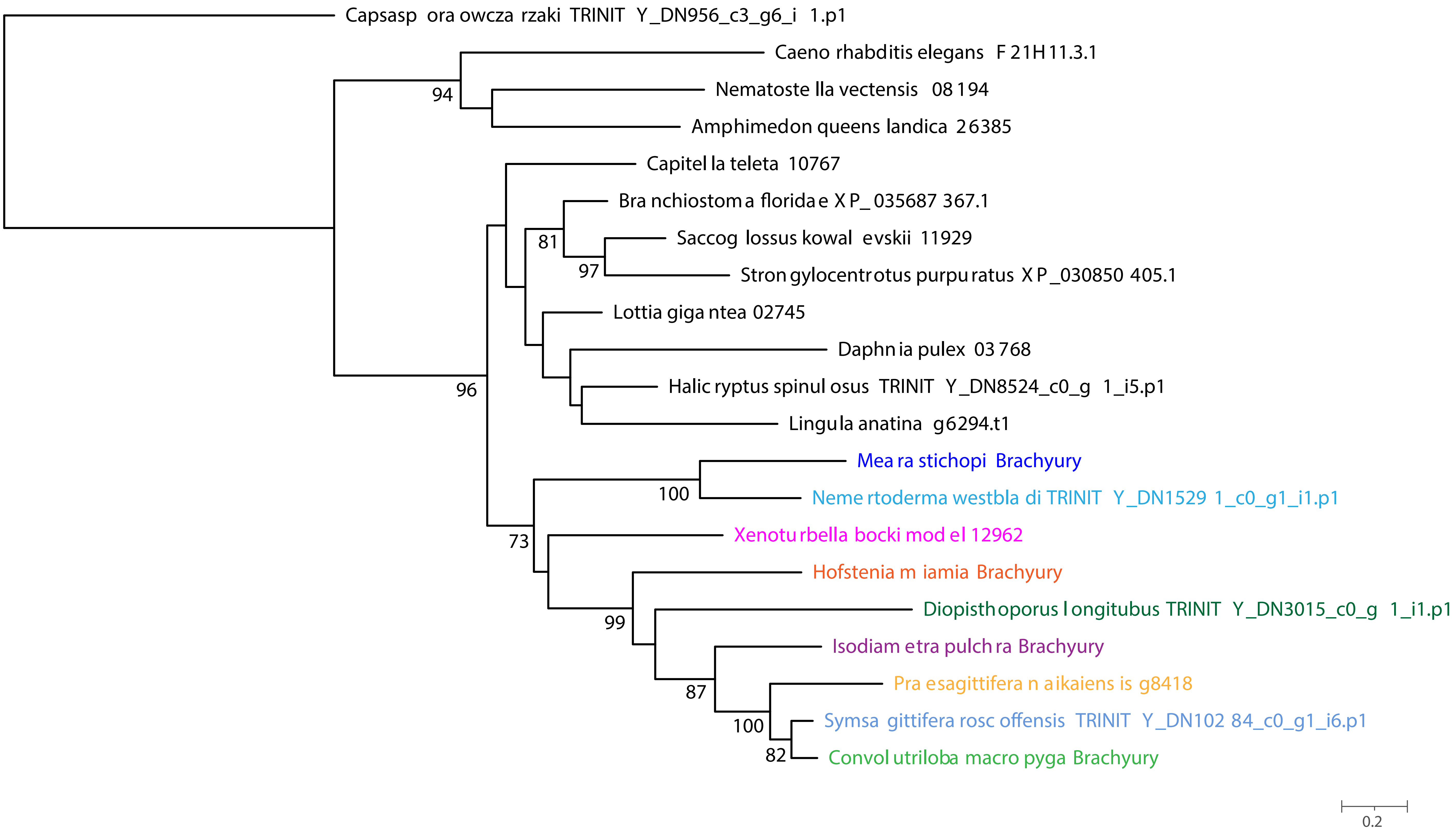

Supplementary Figure 8
Phylogenetic analysis of Cdx sequences

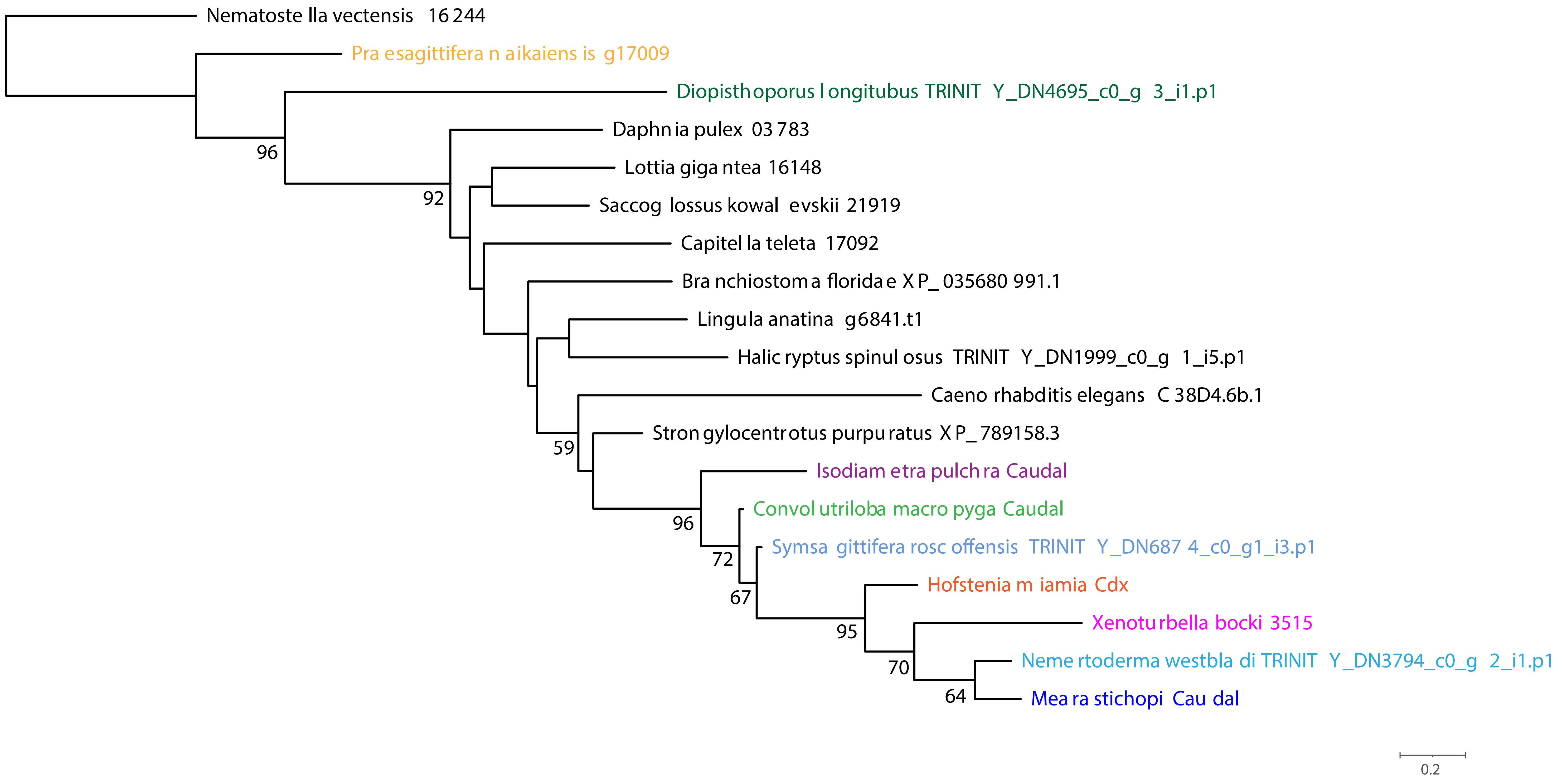

Supplementary Figure 9
Phylogenetic analysis of Evx sequences

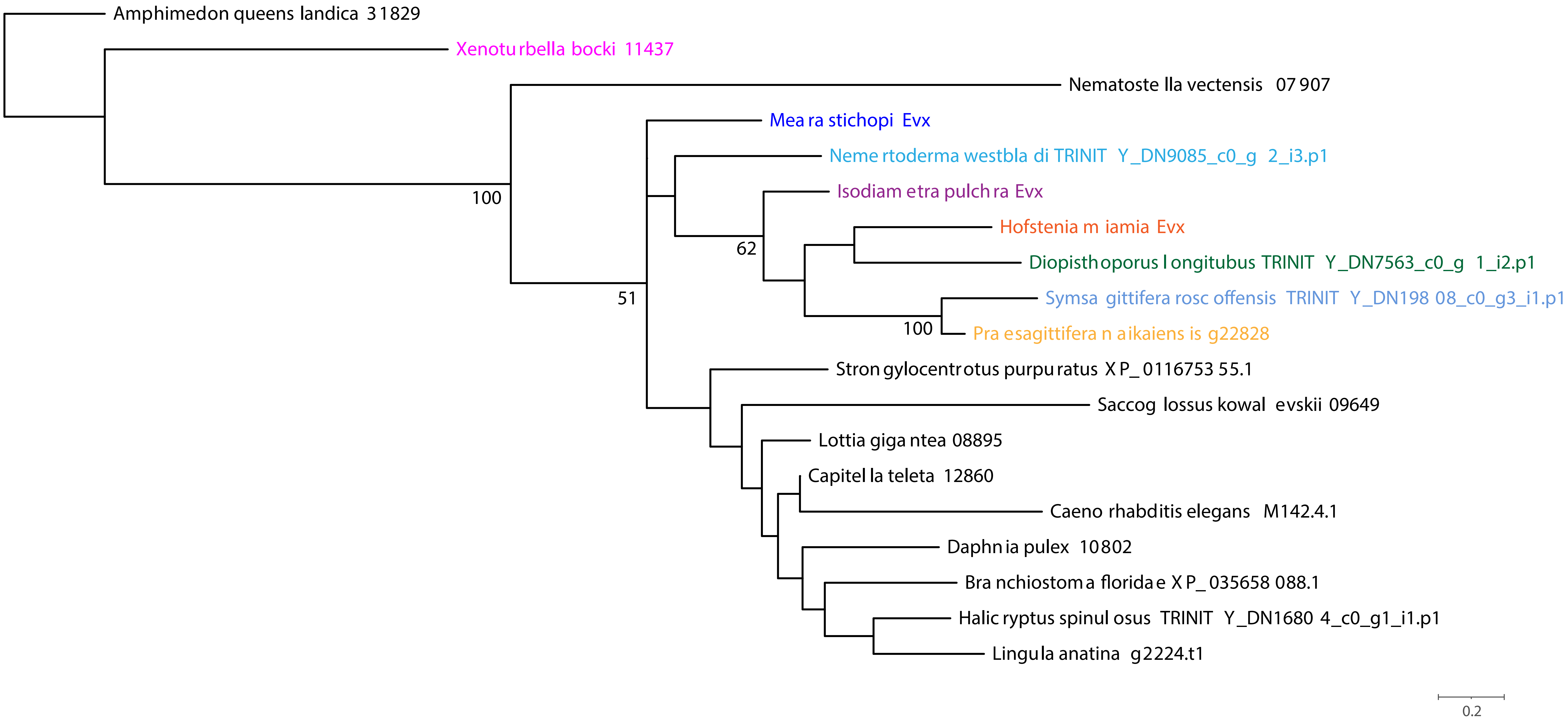

Supplementary Figure 10
Phylogenetic analysis of FoxA sequences

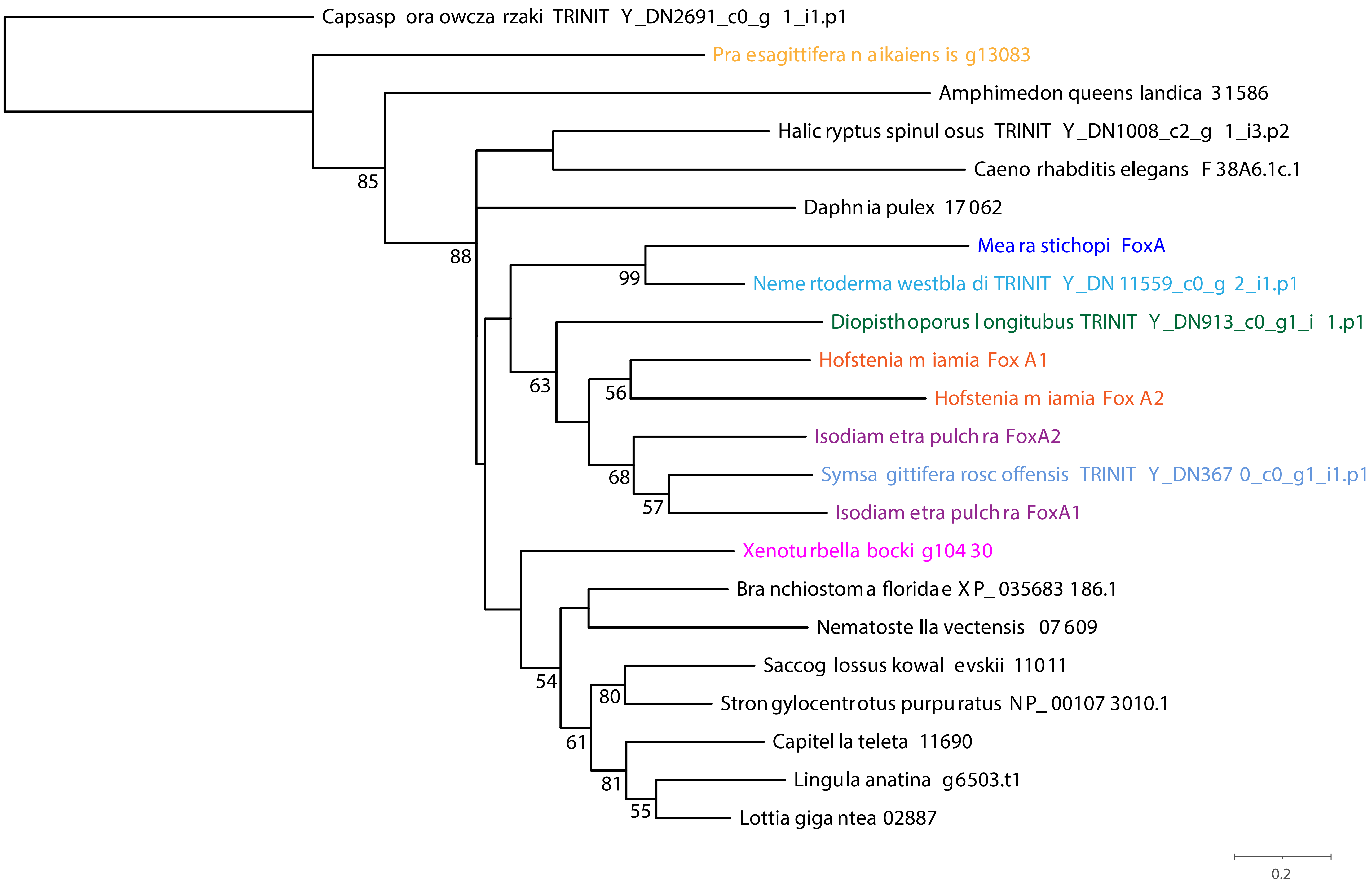

Supplementary Figure 11
Phylogenetic analysis of Frizzled sequences

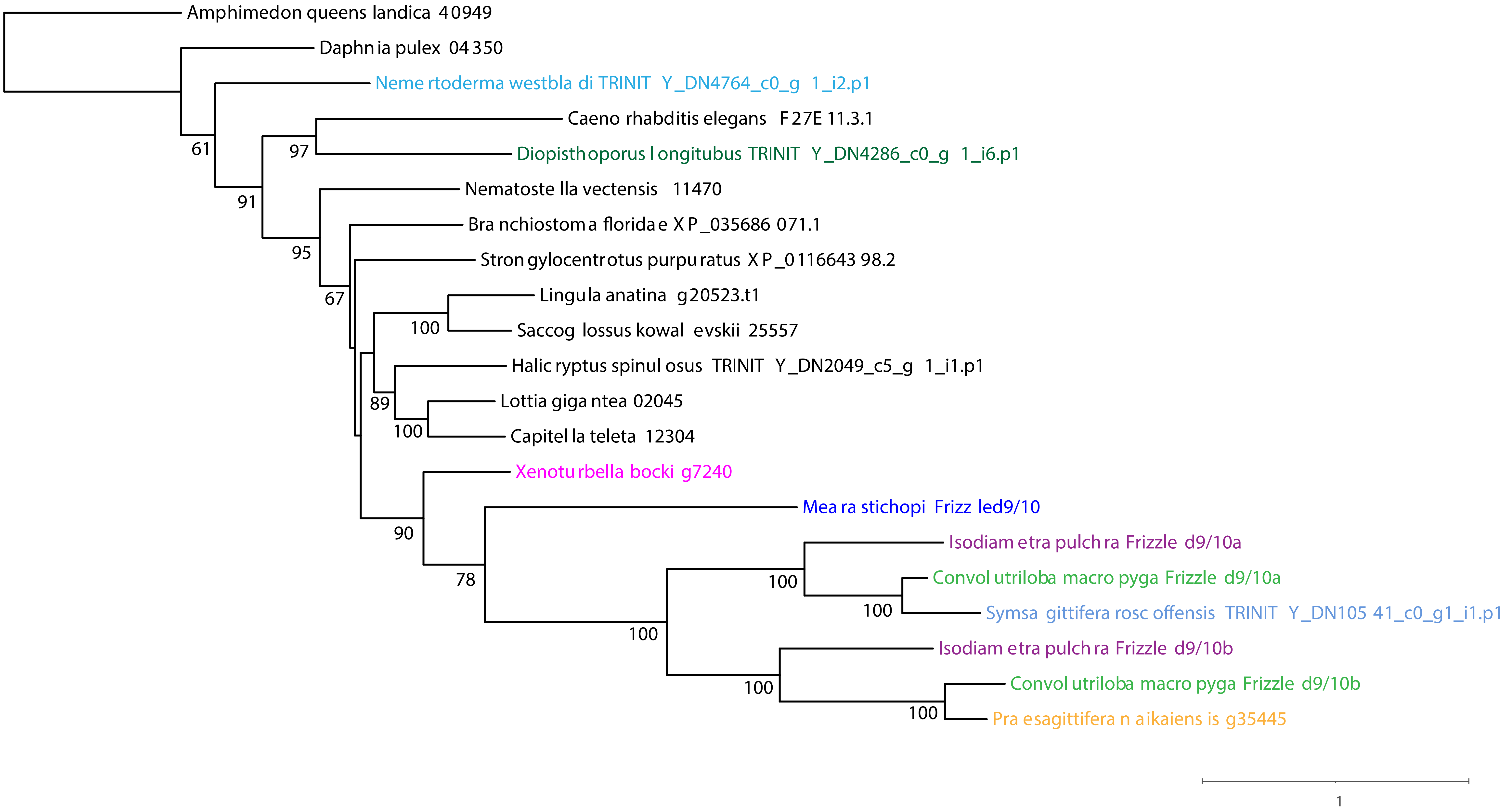

Supplementary Figure 12
Phylogenetic analysis of Gata456 sequences

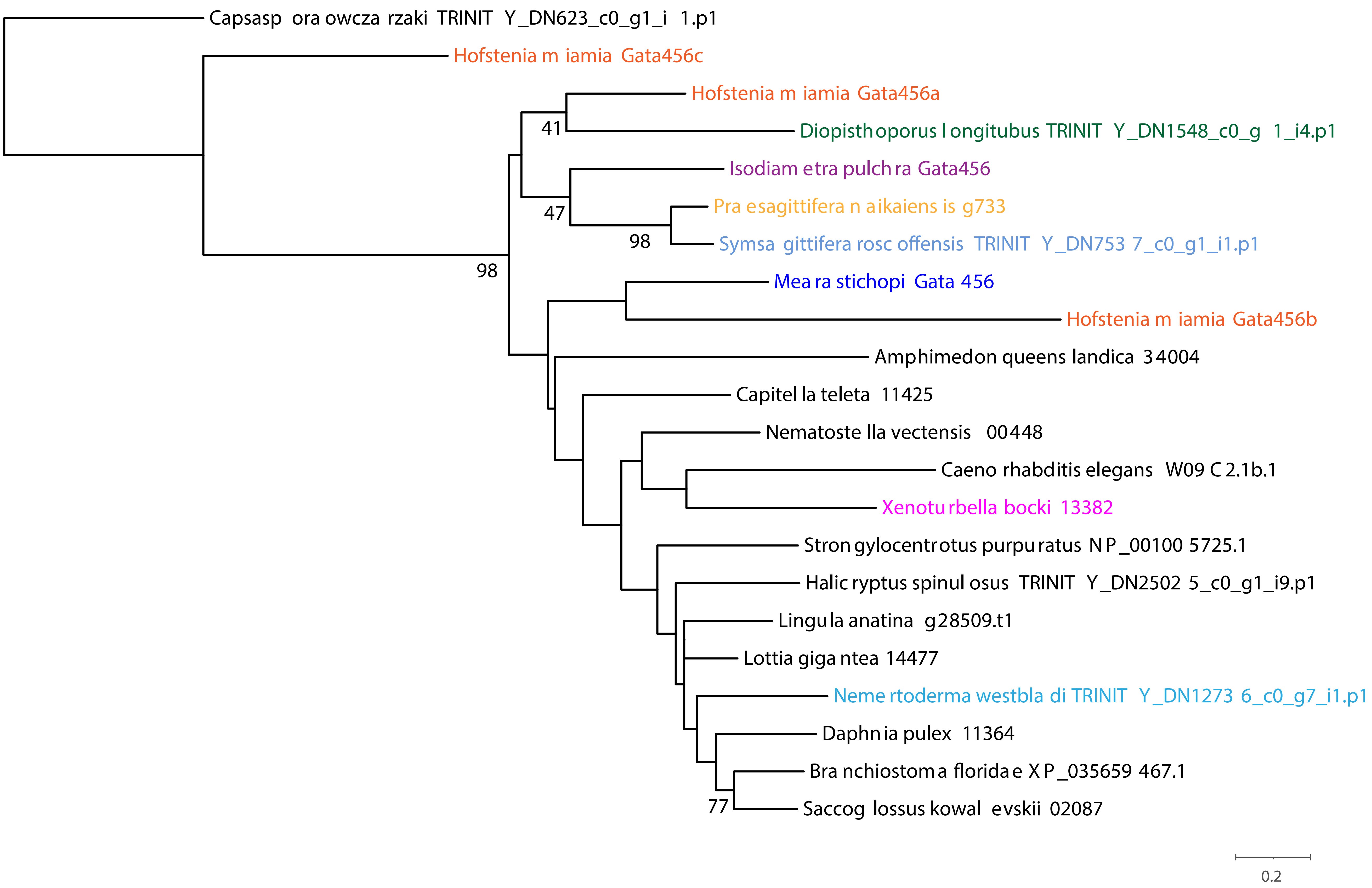

Supplementary Figure 13
Phylogenetic analysis of Goosecoid sequences

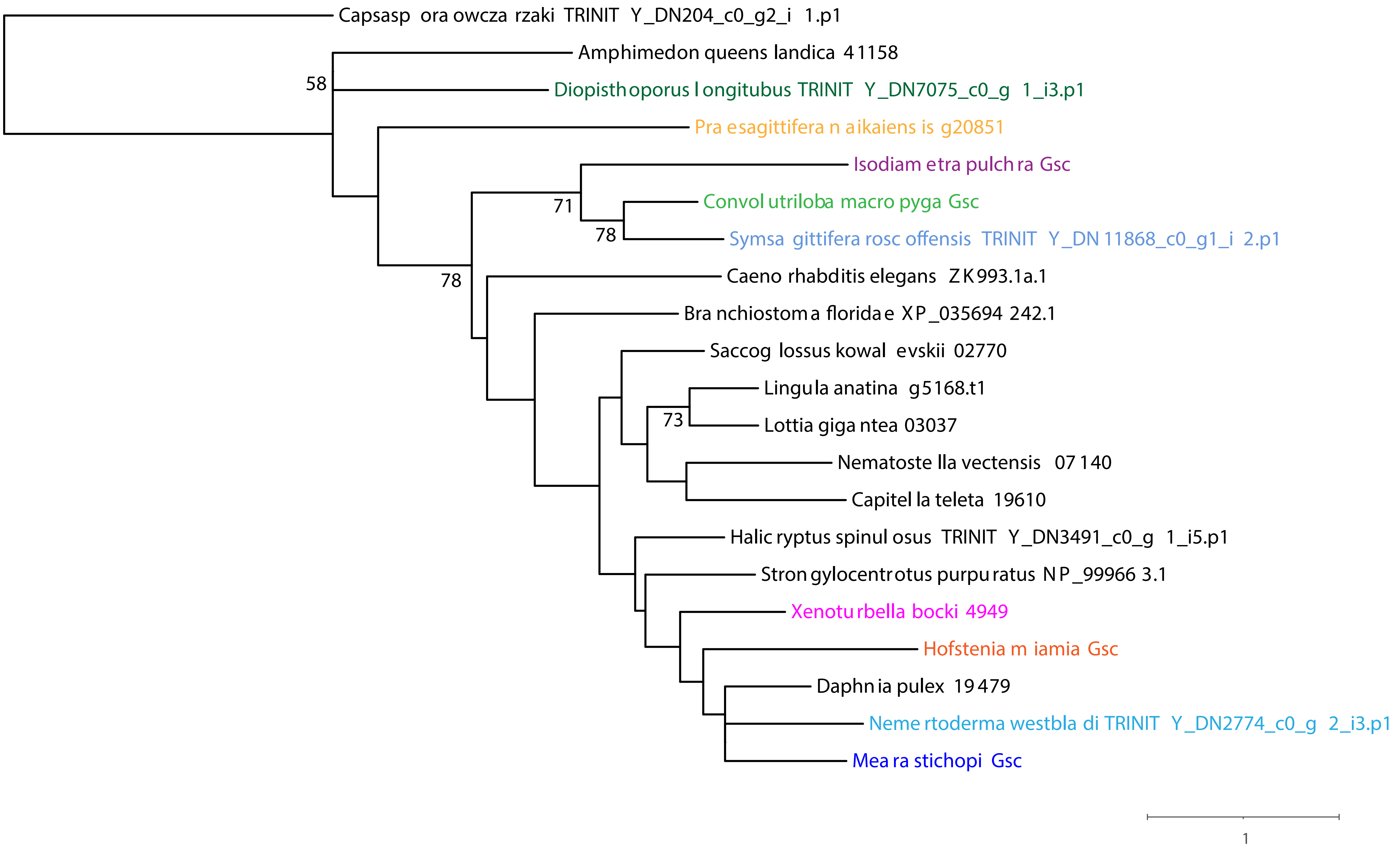

Supplementary Figure 14
Phylogenetic analysis of Hnf4 sequences

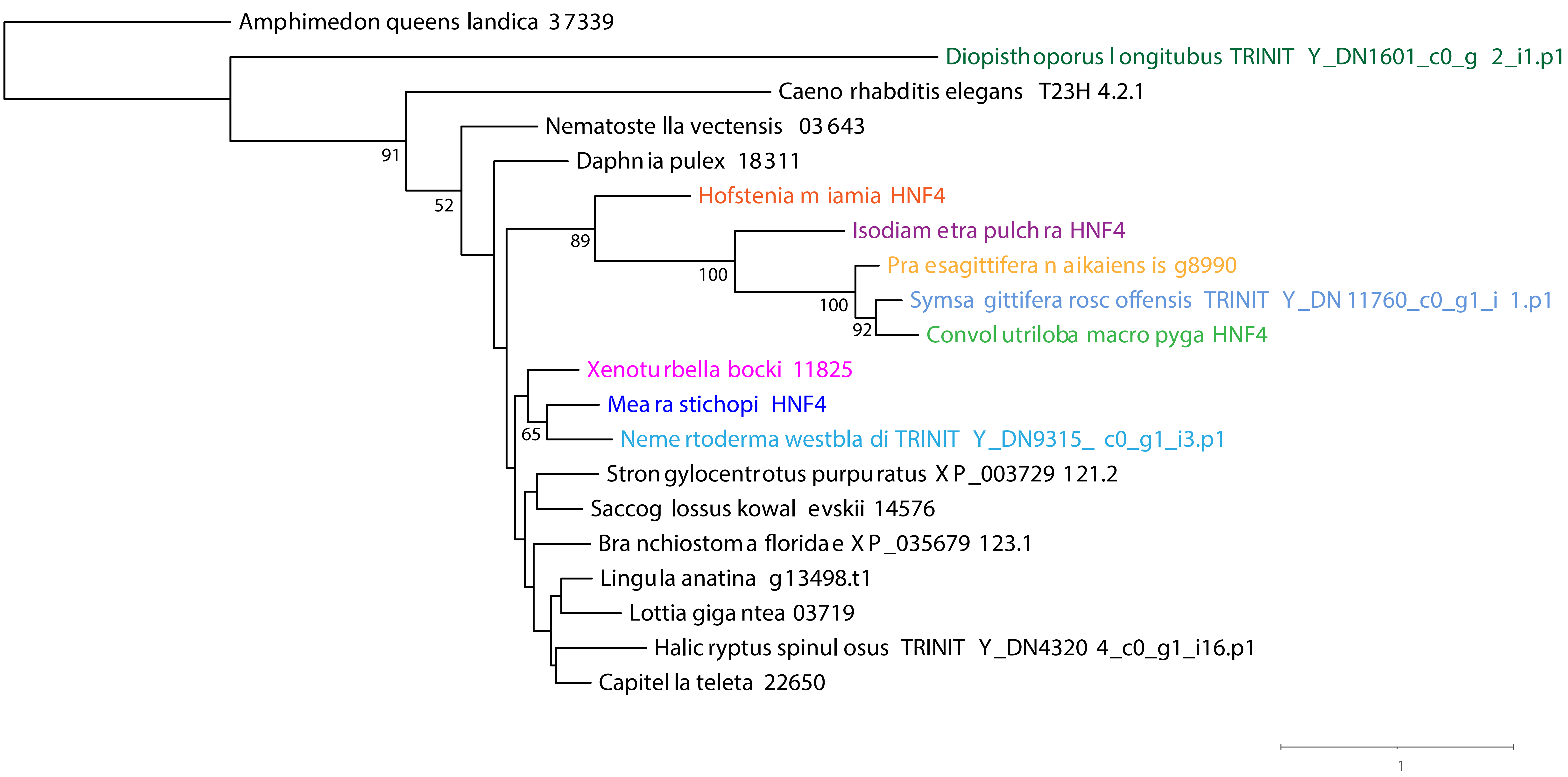

Supplementary Figure 15

Phylogenetic analysis of Nk2.1 sequences

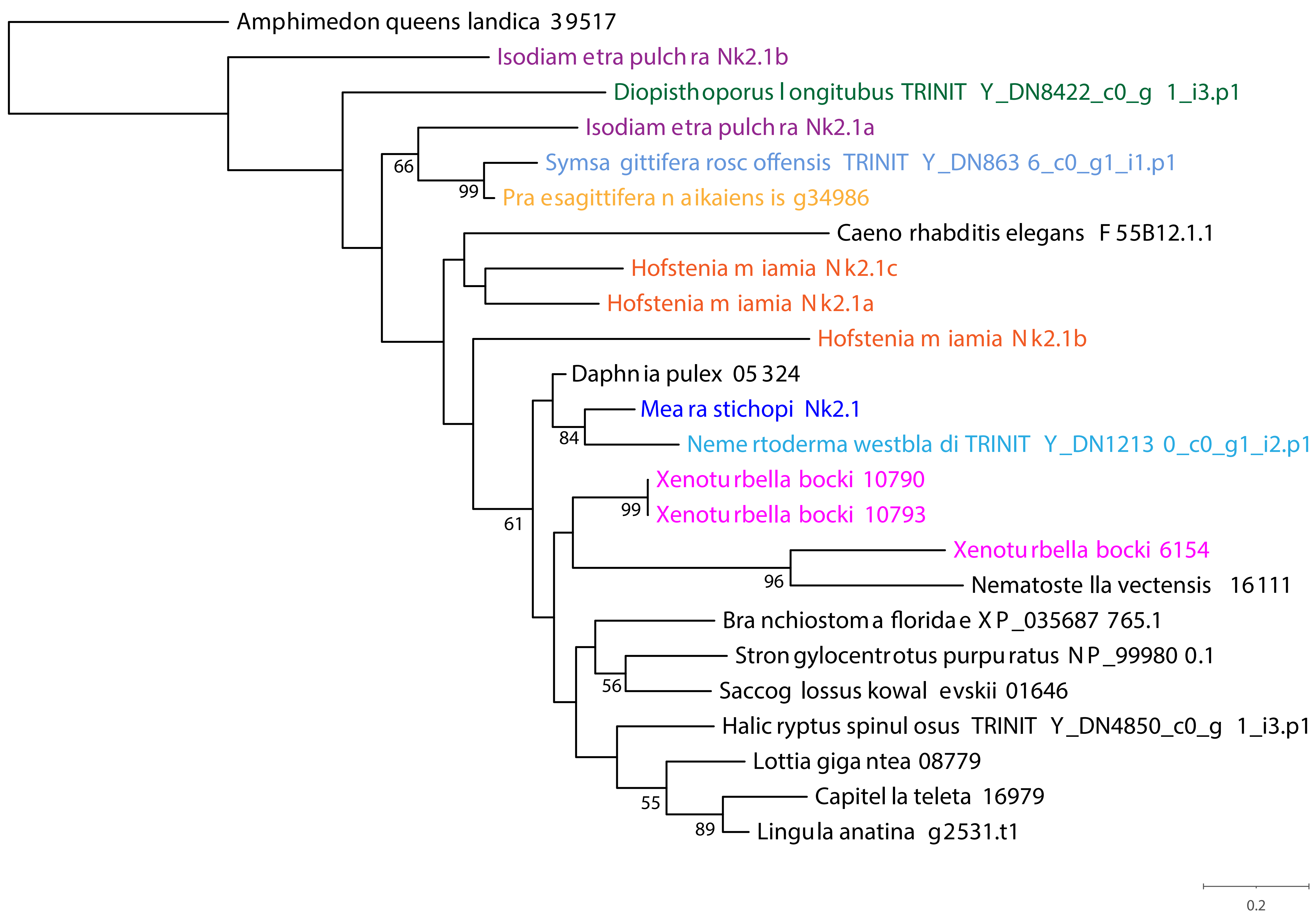

Supplementary Table 1

List of primer sequences used for *Isodiametra pulchra* gene cloning

Isodiametra pulchra	**Primer Forward**	**Primer Reverse**	**RACE 1**	**RACE 2**
Brachyury			CGTGATAAAAGTGCGGGTGACGGGAC	CCCAACTTCGGCTCCCATTGGATG
Cdx	ACCAATCACAACGCAGCATT	TACCCGGTACTTGTCCTTCG		
Goosecoid	TCAATGCGGAGCWCGGCTTSAGTCC	TAGTGGCTGCGGACGAACAC		
FoxA			CTCCAAGGATGGCTGAACCGTTACAAATG	GCTCACTTCCTCCAATGTCCTTCAACAACC
Evx	GCCTCGAGATGGAGTTCAGG	CCAAGTTGCTGCGGAAAGTC		
NK2.1			TCATCATTCTCCCTGACCGACCCCTG	CGCCGCTTCAAGACCCAGAAATACCTGTC
Gata456	AACCCCAAGCAGAGCCTCAG	CGAATTAAGAATGAACCGCAGTG		
Hnf4	CGCAAAAACCACACGTACGT	AATTGCCTGCGACTCCTTCA		
Wnta	ACAACTCTCCTATTTCTCCCCTCAC	ACTTCTCCAGCCGTTCTCTGTAAG		
Wntc	GGACATTTGCCGTTACCTCCTAC	TTCAGTTCCACCCTCTCGTTCACC		
Wntf			GAGCGAGTGAGAATCGAGGATGGGAAAC	TGTTCAAGAAGAGCAGTGGGGAGAAGATCC
Wntd	ACTAAGTGCTGGCTGCGACGGCATC	GGTTGTTGTGGAGGACGATTGC		
Wntb	AACTGCTCGGACAAGTCAGACG	GGAAACGGAATCGTGAAAGAGAAC		
Wnte			ATGCAGTCGTCAAGCCACCACTCGGAG	CGCTGCTGCTCAACAAGTACGACGTG
Wntg	GACCTCGGGAGAGATGAAATGG	GTCAGGAGACGGGTTGATGTAGAG	GCTGCCGTCAATCTCGAAGTGCTCC	GCAGCAGCAACTTCGTTTCTGGCTCG
Frizzled 9/10a	CACCACCAGACAAGTATCAGAGTGC	GCAGAAGCAAGGGACGATGTAAC		
Frizzled 9/10b	TGGAGAAAATGGCTCACTCACTG	GGAGATGGGGAGGATGATGAAATAC		

Supplementary Table 2
List of primer sequences used for *Convolutriloba macropyga* gene cloning

Convolutriloba macropyga	**Primer Forward**	**Primer Reverse**
Brachyury	TCATTGGATGTCGGAGCAGG	GATTGGAAGACGAGGTGGGTAAG
Cdx	CCCATTCGTATCTGGCAGTAGC	GTAGGCTGAGGTGGTTTGTGTTATC
Goosecoid	CATCAACACCAACAGACCATGC	TTGAGTAAGGCTCGCAGAATTACC
Hnf4	GGCGATCGGCAAAAATTCGA	TTGATCGTCCAAGTTGCCCA
Wntb	TTGCTTTCTGCTGGAATCTTGC	GGGTGGTGTGTAGTTGTAGCCATC
Wntd	CAAACATCGGCATTCATCATCC	GACATTCGGCTTCGGAGTCC
Wnta	TTGAGGAACACGCACACAGGAAGC	ACCATCGTCTAATCTTGAACCGAC
Wntf	AGCAGACATTACAAGAGACACGGAC	GGTGACAGCATAGTCGGAGGAATC
Wnte	GTAGAGGCATCCATTTTGGCAC	TCCCACTGTTCTCCTTCTCGTTC
Frizzled 9/10a	CCCGTATCTTCAGTTTTACCCTCAG	ACACCACACCTGTCCATCAACG
Frizzled 9/10b	CGTTATTGTAAGCGGGTGACATTG	CGTCTGTTTGTGCTATTTCGTCC

Supplementary Table 3
List of primer sequences used for *Hofstenia miamia* gene cloning

Hofstenia miamia	**Primer Forward**	**Primer Reverse**	
Brachyury	ATCCCAGCGGACAAAAAGCGATGG	GGGACGGAACGATGTGATTTCAGACGAC	
Cdx	CACTGGACACCATGATGCCT	TTCCAAGATCAAGGGCGCTT	
Goosecoid	TTGTCGCCTCGTTTGTAGCT	TGTACGGTGTCTTCGAGTGC	
Hnf4	GGAGCCCATTGTTTCCGATC	TGCTGCCACCATAAATGTGC	
GATA456a	CAAAAGTCGAGTCAGGCCAC	GCCTAAGAGTGCATGGGTTC	
GATA456b	TTTGCTTGGTAAACAGCTCTG	ATTGCCTTTTCGCCTTCTGG	
FoxA1	CGTTGTTGGAGGTGCAGATT	GGGTTGCTTCATCAGACTGC	
FoxA2	CTACACAGCAGCTTCGACTC	TCGCATGAGGTTTGTGATGT	

Supplementary Table 4

List of primer sequences used for *Meara stichopi* gene cloning

Meara stichopi	**Primer Forward**	**Primer Reverse**	**RACE 1**	**RACE 2**
Brachyury	CAACTTTGGCACACATTGGATGAAGGAGTC	GCGATGAATTGGGTGTCCGTGAAACG		
Cdx	TCAGAGGGCTGAGTTGGAAAATGAATTTCG	TTTCACCTGACGTTCTGAGAGACCGACC		
Goosecoid			GCGGCGATTTTTGAACCATACCTCTACTCG	CTTTCAATGCCAGTTGTTCTCGCAGGAC
FoxA			CCTTTTCCAGGTATGGTGACCCTGAACGAG	CAGCAACGGTGGCAGAACAGCATACG
Evx			GCGTGACCAATTAAACAGACTAGAAAAGG	CTGTGAGTTGGCTGCCCTGTTGAAG
NK2.1			CCAGGTCAACAACGACGAAAGCGTAGAGTG	GACGCCAACGCAGGTCAAAATATGGTTC
Gata456	GTTGTGGCGACGTAACGACAG	TGGGAAGATAAATGGTTTGTGGTG		
Hnf4	ATTCCTGTCGTTTCCAGCGT	AGTTCGCCAAACCTACCTCG		
Ptfa1	GGAAGAGGAGAACACGGTGG	TTCTTCCGGGCTTCGTTCAA		
Wnt11	TAGACCGCCATCAAAGTG	CGATAGCGATTGGACTACTC		
Wnt5			ATATCTGCGGGCAGCTCTCCTGGGTCACTC	CGACCTGTTTGATCTCCCTGCACGTCTC
Wnty	TGCACAAAGATTAGGTCGGTCGGATCTGAC	GGGGCAGGTTACTGGCATTTACTGACATTG		
Wnt1	TGTGAGAGAGAACCAAGGAAGTCTG	CGGCATCTGATGATGAGGGTAG		
Wnt3			TGCAGACATGCTGGATGCAAGTCAGTG	TCACTGACTTGCATCCAGCATGTCTGC
Wntx			GGGCTGCACTTCACAGAGTTTGAAAGACAC	CATCGAGCAGTTGCAGGTTGAAGGTATCG
Wntz			CGATTTCACAGAAGGTAGGAAGGATGGCAG	CAGTTCCGCATGTGCATGAAGAAAACG
Frizzled 9/10	ATTCATTGGTTGTCTGGGCAAG	AAGTCAGGGTTAGCATCGTCCAC		
